# Motivating factors and barriers to help-seeking for casino gamblers: results from a survey in Swiss casinos

**DOI:** 10.3389/fpsyt.2023.1128291

**Published:** 2023-05-25

**Authors:** Suzanne Lischer, Jürg Schwarz, Hannes Wallimann, Emilien Jeannot, Jacqueline Mathys

**Affiliations:** ^1^Lucerne University of Applied Sciences and Arts, Lucerne, Switzerland; ^2^Centre Du Jeu Excessif, Addiction Medicine, Lausanne University Hospital, Lausanne, Switzerland; ^2^Institute of Global Health, Faculty of Medicine, Chemin de Mines, Geneva, Switzerland

**Keywords:** problem gambling, help-seeking, casino, exclusion, public health, disordered gambling, gambling-specific help service

## Abstract

**Introduction:**

Gambling can have serious consequences for many aspects of a person’s life. Yet relatively few people with gambling problems seek help. This study examines the extent to which exclusion from casino venues among other factors may act as a motivator for further help-seeking among casino gamblers (both landbased and remote) with at-risk or disordered gambling behavior. In addition, the barriers that prevent gamblers from accepting help are examined.

**Methods:**

Gamblers from Swiss casinos completed a written questionnaire twice, at 6-month intervals. The questions included whether they had sought help in the past 6 months.

**Results:**

For those with a SOGS-R rating of 1 or over (*n* = 173) at the second survey point, a difference in help-seeking was found between the excluded and non-excluded gamblers (*p* < .001), suggesting that exclusion may be a motivator for help-seeking. Reported differences in levels of debt (*p* = .006), recognition of gambling problems (*p* = .010) and severity of gambling-related problems (*p* = .004) can be taken to suggest that other motivating factors may also influence help-seeking behavior. With regard to the support sought, the most frequently used forms of support were specialized addiction counseling centers (39.5%), followed by self-help groups (21.1%) and remote counseling centers (10.5%). In terms of barriers, reasons relating to attitude, such as denial, appear to pose greater barriers than treatment-related concerns.

**Discussion:**

From a public health perspective, an overarching strategy is required to increase the share of help-seekers among casino gamblers through targeted measures.

## Introduction

Gambling is defined as an activity that involves placing something of value at risk in the hopes of gaining something of greater value. Popular forms of gambling include casino gambling (involving table-based forms, such as blackjack, and electronic-based forms, such as slot machines), lotteries and sport betting and Internet gambling (including poker or sports gambling) (1). It is a common activity across cultures, which for some individuals can evolve into a gambling disorder (GD); a psychiatric condition characterized by persistent, recurrent maladaptive patterns of gambling behavior. GD is associated with impaired functioning, reduced quality of life, high rates of bankruptcy, divorce, and incarceration (2). Globally, past-year prevalence rates for problem gambling have been reported to range from 0.12 to 5.8% (3). Despite the serious consequences, impacting upon many aspects of life, relatively few people with gambling difficulties seek help for their problems (4–6). Estimates of help-seeking prevalence reported in the literature vary substantially. This is due to methodological differences, such as the populations surveyed for help-seeking (e.g., the general population, regular gamblers, or only people with a GD), the types of help sought (e.g., seeking any form of help, or specifically seeking professional help), the time frame reported for GD and help-seeking (e.g., lifetime or current), and geographic differences (7). However, the reasons given in the literature for the low uptake of help offers are consistent with each other. Such barriers to help-seeking can include internal factors such as fear of stigma, shame and denial, individuals` intentions to handle gambling problems by themselves and lack of acknowledgment or minimization of problems followed by concerns about treatment content and quality, lack of knowledge about treatment availability, and practical issues around attending treatment (6, 8–12). External barriers to treatment include lack of awareness of services, difficulty attending sessions due to geographical distance, lack of local specialized knowledge and resources, time constraints, and competing work/domestic demands (10). For migrants, language barriers can be an additional obstacle to help-seeking (13). In addition to the barriers that prevent gamblers from seeking help, several studies have also examined the factors that motivate gamblers to do so. The most important reasons for seeking help appear to be financial pressure and concerns about mental health problems as well as negative emotions (9–11, 14, 15), concerns about the effects of gambling on significant relationships and physical health issues (9, 14). Own ‘recognition of gambling problem (e.g., had reached rock bottom) also seems to be a motivator of help seeking (9). Further findings suggest that recognizing the need for help with gambling problems and making the decision to seek treatment are influenced by demographic factors such as age, gender, ethnicity, and level of education; and attitudinal factors such as perception of the helpfulness of services, perceived stigma, shame and health literacy (8). However, the results of a study related to the use of professional sources of help (problem gamblers) showed no significant differences in terms of the demographic variables gender or age (10). Results from empirical studies on the reasons why gamblers with at-risk or disordered gambling behavior attempt to resolve their gambling problem through gambling specific help service have also been summarized in literature reviews (12, 16).

Formal treatment, however, is not a prerequisite for resolution, even among gamblers with severe problems (17). Recovery rates among gamblers tend to be considerably higher than treatment rates (16). Since treatment options for GD may include both professional interventions within formal treatment systems, as well as peer support interventions (18), it is important to consider alternative pathways to recovery from GD (19). Evidence suggests that gamblers engage in a broad range of self-management actions and activities to improve their symptoms of problem gambling, as well as seeking help from distance-based services (e.g., helplines) and family and friends (15). Furthermore, there is evidence that mutual support societies for problem gamblers (20) or self-help groups may constitute an important option for those suffering from GD (21). As stigma has been identified as a major barrier to help-seeking, treatment and recovery from gambling problems (22), it is also possible that individuals prefer to seek help from general practitioners (10) or services outside addiction counseling, such as debt counseling centers (23). Self-exclusion from casinos can be considered as a form of help-seeking since the gambler is approaching an external source for assistance in grappling with the problem (16). However, it should be emphasized that self-exclusion does not constitute a formal treatment intervention but rather presents an opportunity for immediate assistance to limit further financial losses by blocking direct access to gambling opportunities (24). Nevertheless, researchers assume that self-exclusion-programs provide an opportunity to serve not only as a preliminary barrier to gambling access but also as an effective gateway to further treatment services (24, 25). Thus, it can be assumed that exclusion, whether imposed or voluntary, is a plausible motivator for entering treatment.

Within this context, the present study examines how exclusion may act as a motivator for help-seeking among casino gamblers (both land-based and remote) who present with at-risk or disordered gambling behaviors. However, there are other drivers that motivate individuals with a problematic gambling behavior to seek help. As mentioned above, research has already identified several motivating factors that may serve as driver for seeking help. Against this background, the present study considers also the socio-demographic parameters age and gender, severity of gambling disorder, mental health problems, debts due to gambling, relationship issues, and one’s own and others’ perceived gambling-specific problems as possible motivators for seeking help. In addition to the question of what contribution exclusion and other motivators are likely aiding in the uptake of gambling specific help services the study seeks to determine which offers of help are used by persons seeking help, and why individuals with problematic gambling behavior may have so far refrained from seeking help. Overall, the study aims to better understand factors that motivate casino gamblers to seek help or hinder them from help-seeking. The results are intended to provide public health practitioners with an empirical basis for planning and implementing tailored help services.

### Setting

In Switzerland, gambling is regulated by the Federal Gambling Act (26). The total of 21 casinos within the country offer a range of table games, slot machines and poker. Since January 2019, land-based casinos can apply for a license extension for online casino games. The gambling market is also supplemented by land-based gambling offers which are located close to the border in neighboring countries, and the online gambling offers of international gambling operators (27). In addition, sports betting and lotteries are easily accessible, being sold through kiosks and newsagents, throughout the country (28). A population survey conducted in 2017 found that 12-month gambling prevalence was highest for Swiss lotteries (48.2%), with 8.6% reporting having bet on table games in casinos and 6.7% slot machines. At the time of this population survey, legal online gambling was not yet available. Therefore, the 2.3% of respondents who stated that they participated in online gambling did so at a time when providers were not yet licensed in Switzerland (29).

The Federal Gambling Act requires every casino and the two lottery companies to develop a clear player protection strategy. Bans are imposed if proof can be found that, due to their gambling behavior, specific gamblers are maintaining excessive debts, placing bets that are disproportionate to their financial circumstances, or experiencing other disruptions. On the other hand, gamblers can also ask to be self-excluded. An exclusion is of an indefinite duration, but after a minimum of 3 months, a revocation may be requested (30). The exclusion program covers multiple venues, i.e., a banned gambler is excluded from land-based casinos and licensed online-casinos as well as online lotteries and sports betting (27). A total of 12,133 new exclusions were issued during 2021, bringing the total number of exclusions in effect nationwide to 79,917, at the end of 2021 (31). Casinos as well as lottery providers are obliged by law to embed their social protection measures and the corresponding activities at the cantonal level, which in concrete terms means that the providers have a specialized addiction center as a regional partner (26).

Within the Swiss population, the extent of GD was measured by the National Opinion Research Centre Diagnostic and Statistical Manual of Mental Disorders (NODS-CLiP), was estimated to be 2.8% for at-risk gambling behavior and 0.2% for disordered gambling behavior, in 2017 (29). The estimate of an institution survey conducted throughout Switzerland concluded that on the survey date (in mid-March 2022), 216 persons were in a medically oriented institution and 408 persons were in a psychosocially oriented institution seeking outpatient treatment or counseling, with GD as their main problem. At the same time, a further 13 persons were in inpatient treatment for GD (32). Generally, around 15% of the Swiss population felt moderately to severely psychologically stressed in 2017. When asked specifically about depression symptoms, around 3% reported (rather) severe, 6% moderate and 26% mild symptoms. The proportion of the population that sought outpatient treatment for psychological distress was just over 6% in 2017 (33). Since the beginning of the Covid-19-pandemic, more people have reported increased psychological distress (34). Regarding gambling treatment or counseling programs, most of these are incorporated into mental health treatment facilities and are typically provided by institutions that treat substance-use disorders. Only a few institutions offer specialized services for people with GD. Treatment costs are covered by universal mandatory Swiss health insurance. Intervention programs, delivered mainly from an individual or group approach, have proved useful in the treatment of GD and its psychiatric comorbidities (35).

## Method

### Procedure

The present study is part of an ongoing research project in which excluded and non-excluded gamblers are interviewed about their gambling behavior, and about their motivations to seek help at 6-month intervals. The respondents were recruited from 19 of the 21 Swiss casinos. As a first step in the recruitment process, the consent of the casino directors was obtained. The persons responsible for player protection in the respective casinos, who from then on acted as a point of contact for the study, were informed about the study in a briefing session. As a subsequent step, the persons responsible for player protection instructed the casino employees, who recruited the study participants from the land-based casino, either on site with the help of flyers or online by emailing the flyers. A separate flyer was created for the recruitment of the non-excluded gamblers. The flyer contained a link as well as a QR code to the website set up especially for this purpose, through which the study participants received the study information, gave their consent to participate in the study, and could register for study participation with their e-mail address. The electronic questionnaire, generated with Unipark online survey software, was sent to the participants by email. The excluded gamblers took part in the first survey immediately after their exclusion entered into force. The non-excluded gamblers were given the ongoing opportunity to sign up for the study. Six months later, they received an e-mail for the second survey. Participation was rewarded with a shopping voucher of 20 Swiss francs. Completed questionnaires that could be assumed to be cheating (for example, those that had taken an unrealistically short time to complete) were removed from the data set used for the analyses. The data was stored on a secure database at the Lucerne University of Applied Sciences and Arts. The survey lasted from September 2019 to January 2022. It was conducted in the three language regions of Switzerland (German, French and Italian). Participants were not asked to report which casino they played in most often, so the level of representation for each of the 19 participating casinos, is unknown.

### Measures

#### Demographics

Standard questions were included, to collect data on gender and age.

#### Exclusion

Participants were asked if they were excluded from gambling participation. A distinction was made as to whether the exclusion was voluntary or imposed and whether land-based or online gambling was the determining factor for the exclusion.

#### Problem gambling

The South Oaks Gambling Screen (SOGS) is a 20-item instrument used to screen for pathological gambling (36). The SOGS-R is scored by summing the number of items endorsed, out of 20. A cut-off score of five or more indicates that the respondent is a probable pathological gambler, whereas a score between one to four indicates some gambling problems. The answers refer to the last 6 months (37). Authorized German, French and Italian versions were used for the survey (38–40).

#### Mental health

Mental health concerns were investigated using the four-item Patient Health Questionnaire (PHQ-4). The PHQ-4 consists of two subscales, each containing two items for depression and anxiety with scores ranging from 0 to 6 points for each subscale (41). The PHQ-4 consists of the first two items of the Generalized Anxiety Disorder scale (GAD-7) (42) and the first two items of the Patient Health Questionnaire (PHQ-9) (43). Respondents rate their symptoms using a four-item Likert rating scale ranging from 0 (not at all) to 3 (nearly every day), and the total score ranges from 0 to 12. The severity of clinically relevant depression and/or anxiety according to the PHQ-4 score is to be interpreted as follows: none to minimal (0–2), mild (3–5), moderate (6–8), severe (9–12). For the German, Italian and the French versions of PHQ-4 the instruments PHQ-9 and GAD-7 were taken from Pfizer (44).

#### Current and previous use of supportive treatment or counseling services

The self-constructed question “*Have you ever used supportive treatment or counseling services because of gambling*?” could be answered with “*yes*,” “*no, not yet*” or “*no, because I have no problems*.”

#### Gambling specific help services

Respondents who answered in the affirmative to the previous question about whether they had used help, were asked about the type of gambling-specific help service(s) they had used (a) in the past 6 month or (b) in the period prior to that. In the analysis, the responses that referred to the last 6 months were considered. The options were as follows; *self-help-groups, online-self-help-groups, debt counseling, general practitioner, psychotherapist or psychiatrist, addiction treatment inpatient services, addiction counseling, online counseling, significant others, religious dignitaries, others*. Categories with a low number of mentions were then aggregated, for statistical purposes and a final list can be seen in Table 4.

**Table 4:** Use of gambling specific help services (SOGS-R ≥ 1, *n* = 23, multiple choice).

Self-help groups	34.8% (8)
Online self-help groups	26.1% (6)
Counseling services, psychiatrist or psychotherapist	65.2% (15)
Online counseling services	17.4% (4)
Debt counseling	8.7% (2)
Addiction treatment inpatient services	4.3% (1)
Significant others	17.4% (4)
Other	4.3% (1)

#### Barriers that prevent gamblers from help-seeking

Those gamblers with a SOGS-R-score ≥ 1, who indicated that they had “not yet” sought help were asked about the reasons why they had not yet done so. The altogether 14 possible reasons listed, were taken from the questionnaire of the German study “Risk and protective factors for overcoming gambling problems” (45). Of these, 10 questions were taken from the “TACOS-General Population Study” (46). The questionnaire was translated and back translated in French and in Italian. Subsequently, the questions were checked by native-speaking psychologists.

#### Further motivators for using gambling specific help services

Other factors that may influence the motivation to seek help were investigated, as follows: Debts due to gambling (*do you have debts due to gambling?*); own recognition of gambling problems (*do you feel you have ever had a problem with betting money or gambling*?); gambling problem recognized by others (*have people criticized your betting or told you that you had a gambling problem, regardless of whether or not you thought it was true*?). The last two questions were taken from the SOGS-R (37). Another question was asked about whether the individual had been made aware of treatment or counseling services by a casino staff member (*have you been referred to treatment or counseling services by a casino staff member?*).

#### Six-month gambling prevalence

To measure gambling behavior, the questionnaire contained questions on respondents’ use of the different types of gambling products available in Switzerland and abroad during the past 6 months. A total of 25 game-categories were surveyed, which were condensed to a total of six categories for statistical analysis.

## Statistical analyses

Statistical analyses included basic statistics (mean, etc.) and statistical tests (chi-square test, Fisher’s exact test). Statistical significance level was set at α = 0.05. Analyses were performed using the statistical software R.

### Compliance with ethical standards

The Swiss Ethical Authority decided that the project did not require formal ethical approval since it does not involve research on human diseases or the structure and function of the human organism (file number Req-2019-00060). The participants provided their written informed consent to participate in this study. The data management plan was approved by the Swiss National Science Foundation.

The data, provided by study participants, are stored on a secure server at the Lucerne University of Applied Sciences and Arts. On behalf of the Swiss National Science Foundation, a data management plan was created, to specify exactly how data security is guaranteed.

## Results

### Characteristics of the baseline study sample

Table 1 displays the characteristics of all respondents who participated in the first survey.

The survey is part of an ongoing study that examines the impact of exclusion on various indicators, including motivation to seek help. Since excluded gamblers were targeted for recruitment in the study, this sub-group is overrepresented in the sample (accounting for 24.8% of participants), and consequently, the proportion of gamblers with GD is also overrepresented. In the baseline survey, 12.4% reported seeking help for gambling-related problems in the past 6 months.

In the baseline survey, 36.9% of casino gamblers reported having no gambling-related problems, 42.8% had some problems, and 20.4% can be considered “probable pathological gamblers” according to the SOGS-R. There is a significant difference between excluded and non-excluded gamblers in this regard (*p* < .001). Among the excluded gamblers, the percentage of those who reported having no problems according to SOGS-R is 8.3%. 42.9% had “some problems” and 48.8% can be considered “probable pathological gamblers.” Among non-excluded gamblers, 46.3% had no problems, 42.7% had some problems, and 11.0% were likely to have pathological gambling behavior. Only 8.2% of the respondents claimed that they had debts due to gambling. 28.8% reported having no or minimal mental health problems, 24.5% showed mild problems. However, a total of 29.8% had moderate mental health problems, and 16.9% had serious mental health problems according to PHQ-4. With regard to support, 9.5% of the respondents indicated that they had been made aware of addiction counseling or treatment services by casino employees. Furthermore, 24.5% of respondents reported that significant third persons had identified them as having a gambling problem, whereas 27.7% of respondents recognize their own gambling behavior as problematic.

In terms of 6-month gambling participation, respondents could indicate several forms of gambling. Land-based table games in casinos were mentioned most frequently (84.4%). It is noticeable that fewer gamblers (32.4%) participated in licensed online games than in unlicensed (illegal) offerings (39.2%). In addition, 36.3% of gamblers reported using land-based lotteries and sports betting. We should note that, unlike corresponding online offers, land-based lottery products are not part of the overarching exclusion system.

### Characteristics of the gamblers: help-seekers versus non help-seekers

Six months after the initial survey, gamblers were asked about the same parameters. A total of 269 gamblers responded at the second time point of the survey. Of these, 11.1% (*n* = 30) had sought help and 88.8% (*n* = 239) had not. Thus, the drop out of respondents since the first survey time point amounts to 70 individuals. The analysis of these shows that the gamblers who dropped out do not differ from the study population. For example, 9.5% of these gamblers reported seeking help for gambling-related problems (baseline: 12.4%, see Table 1). In addition, 68.9% (baseline: 60.3%) are men, and 58.1% (baseline: 58.5%) are between 26 and 45 years old. In addition, 23.0% can be considered “probable pathological gamblers” (baseline: 20.4%). However, there is a slight difference when considering mental problems. 10.8% of the gamblers who dropped out can be classified as having severe mental health problems according to the PHQ-4. In the baseline survey, the proportion of persons with severe mental health problems amounted to 16.9%.

As gamblers who have a SOGS-R score of 0 have no apparent reason to seek help for gambling-related problems, these gamblers were removed from the sample and following analysis (Except Table 3: The influence of mental health on the use of help). The differences between gamblers with a SOGS-R ≥ 1 who had sought help in the last 6 months and those who had not can be seen in Table 2. At the second interview time point, the sample of 173 respondents with a SOGS-R ≥ 1 included 23 persons who had sought help.

**Table 2:** Comparison of help-seeking (HS) versus non help-seeking (NHS) gamblers (SOGS-R ≥ 1).

	HS *n* = 23	NHS *n* = 150	*p*-Value
Sex			.266
Male	60.9% (14)	74.5% (111)	
Age			.961
18–25	26.1% (6)	22.3% (33)	
26–45	60.9% (14)	61.5% (91)	
46–65	13.0% (3)	14.9% (22)	
66 and above	0.0% (0)	1.4% (2)	
Excluded			<.001*
Yes	60.9% (14)	21.3% (32)	
No	39.1% (9)	78.7% (118)	
SOGS-R			**.004** *
Some problem (1–4)	52.2% (12)	81.3% (122)	
Prob. Path. Gamb. ≥5	47.8% (11)	18.7% (28)	
Debts due to gambling			**.006** *
Yes	28.6% (6)	6.4% (9)	
Made aware of treatment by a casino staff member			.961
Yes	13.0% (3)	6.8% (10)	
Gambling problem recognized by others			.068
Yes	47.8% (11)	26.7% (40)	
Own recognition of gambling problems			**.010** *
Yes	60.9% (14)	32.0% (48)	
Gambling			
Land based casino	47.8% (11)	80.7% (121)	**.001** *
Licensed online games	34.8% (8)	36.0% (54)	1
Online Swiss Lotto/Sports betting	0.0% (0)	20.7% (31)	.961
Land-based Lotto/Sports betting	34.8% (8)	35.3% (53)	1
International online games	34.8% (8)	30.7% (46)	.877
Others	17.4% (4)	31.3% (47)	.263

All tests were performed with a chi-square test except when the conditions were not met. In this case, Fisher’s exact test was used.

* *p* < .050.

**Table 3:** Comparison of mental health scores for all help-seeking gamblers versus non help-seeking gamblers (*n* = 256, *n* = 13 missing).

	HS *n* = 28	NHS *n* = 228	*p*-Value
**PHQ-4**			.056
None-to-minimal	7.1% (2)	28.9% (66)	
Mild	32.1% (9)	26.8% (61)	
Moderate	35.7% (10)	28.1% (64)	
Severe	25.0% (7)	16.2% (37)	

The *n* = 28 gamblers who sought help, *n* = 23 have a SOGS ≥ 1 and *n* = 5 have a SOGS = 0.

Table 2 compares gamblers who did or did not seek help, 6 months after the first survey. As mentioned above, the analysis includes only gamblers who have a score SOGS-R ≥ 1, as one can assume that only these gamblers have a rationale for seeking help due to GD. Under these premises, the percentage of gamblers who sought help is 13.3% (*n* = 23). Meanwhile, a differentiation was made between whether the SOGS-R had a score of 1–4 (some problems) or 5 and over (probable pathological gambling). In the category “some problems,” the proportion of those who do not seek help is larger. In the category of “probable pathological gambler,” the proportion of those gamblers who sought help was significantly higher (*p* = .004). In terms of motivation to seek help, a significant difference can be observed between excluded and non-excluded gamblers (*p* < .001). Accordingly, it appears that exclusion is indeed a motivator to seek help. Debt can also be seen as a motivator (*p* = .006); however, the result should be interpreted with some caution, due to the small sample size. Regarding the recognition of gambling-related problems, there is also a significant difference in terms of recognizing one’s own problem (*p* = .010). No difference can be observed between the two groups (help-seekers versus non help-seekers, SOGS-R ≥ 1) regarding significant others who recognized a problematic gambling behavior.The fact that casino employees draw gamblers’ attention to existing offers, however, does not seem to have any influence on the motivation to seek counseling (*p = *.961). In terms of age and gender, no difference can be observed either. Again, the results should be interpreted with caution due to the small number of cases.

With respect to the 6-month prevalence of gambling participation, no difference can be detected regarding the motivation to seek help. Table games are an exception (*p* = .001). The proportion of individuals who do not seek help is significantly higher among gamblers who bet on table games.

Finally, it should be noted that of these 173 respondents, five gamblers (2.89%) have ceased gambling altogether. It is noteworthy that three of these five persons indicated that they had sought help for gambling-related problems in the last 6 months.

### The influence of mental health on the uptake of help

Gambling problems are associated with other mental health disorders including depression, anxiety disorders, and others (47). This association can also be proven in the present study. The significant Spearman correlation coefficient value of 0.289 indicates a weak positive correlation between PHQ-4 and SOGS-R scores, weakly indicating that if the PHQ-4 of gamblers increases, the SOGS-R increases and vice versa. Due to this correlation, all gamblers were considered in the analysis regarding their motivation to seek help (i.e., also those with a SOGS-R score = 0). Otherwise, there would have been a bias, as presumably some of the gamblers with mental health problems would not have been taken into account. The correlation of mental health problems and motivation to seek help is therefore not presented with the other parameters in Table 2 but separately in Table 3. Table 3 thus shows that there is a total of n = 5 individuals who report a SOGS-R score = 0, but who nevertheless sought help. Given the circumstances regarding sample size and achievable power, the analysis shows no clear result with a value of p of .056.

### Type of gambling-specific help service

Table 4 shows the share of gambling-specific help services used by those seeking help 6 months after the first survey. Again, the analysis includes only gamblers who have a score SOGS-R ≥ 1.

**Table 1:** Characteristics of the baseline study sample (*n* = 339).

**Help-seeking**	
Yes	12.4% (42)
Sex	
**Male**	60.3% (240)
**Age**	
18–25	26.6% (89)
26–45	58.5% (196)
46–65	13.4% (45)
66 and above	1.5% (5)
**Excluded**	
Yes	24.8% (84)
**SOGS-R**	
No problem (0)	36.9% (125)
Some problem (1–4)	42.8% (145)
Prob. Path. Gamb. ≥5	20.4% (69)
**PHQ-4**	
None-to-minimal	28.8% (94)
Mild	24.5% (80)
Moderate	29.8% (97)
Severe	16.9% (55)
**Debts due to gambling**	
Yes	8.2% (26)
**Made aware of counseling/treatment services by a casino staff member**	
Yes	9.5% (32)
**Gambling problem recognized by others**	
Yes	24.5% (83)
**Own recognition of gambling problems**	
Yes	27.7% (94)
**Past-6-month gambling prevalence**	
Land based casino	84.4% (286)
Licensed online games	32.4% (110)
Online Swiss Lotto/Sports betting	19.8% (67)
Land-based Lotto/Sports betting	36.3% (123)
International online games	39.2% (133)
Others	43.1% (146)

Outpatient addiction counseling services are the most used form of gambling-specific help service (65.2% uptake). Self-help groups are the second most frequently used help service (34.8%). It is noticeable that remote help services are utilized less frequently. Only two individuals in the sample made use of debt counseling services.

### Barriers to help-seeking

Gamblers who indicated that they had not sought help were able to choose between the answers “no, because I do not have any problems” and “no, not yet.” Those giving the latter response were asked the reason for this. Again, only those gamblers with a SOGS-R score ≥ 1 were considered.

23 (13.3%) individuals reported having used a gambling-specific help service at the second interview time point. 66 (38.2%) of gamblers said they did not seek help because they do not have problems. A further 84 (48.5%) individuals indicated that they had not yet sought help,

An in-depth examination of the group (*n* = 66) who claim to have no gambling-related problems shows that of these 90.9% (*n* = 60) respondents met between 1 and 4 criteria (some problems) on the SOGS-R, while 9.1% (*n* = 6) were probable pathological gamblers.

A further 48.0% of individuals indicated that they had not yet sought help. Respondents from the group that indicated they had not yet sought help were asked about specific barriers that prevented them from seeking help (Table 5). These respondents can be characterized as follows: The proportion of individuals with “some problems” is 73.8% (*n* = 62) and the proportion of persons who are likely to have pathological gambling behavior according to SOGS-R is 26.2% (*n* = 22).

**Table 5:** Barriers that prevent gamblers from help-seeking (*n* = 84, SOGS-R ≥ 1).

	1	2	3	4	5	Missing
	%					*n*
**Treatment related**						
I did not know where to go to get help.	11.2	18.8	25	33.8	11.2	4
I did not believe that treatment would help me.	4.9	17.3	32.1	34.6	11.1	3
I thought that treatment would cost me too much time and energy.	8.5	9.8	26.8	35.4	19.5	2
I have had rather bad experiences with professional offers of support.	20.5	14.1	28.2	24.4	12.8	6
There was no specialized help available for gambling problems where I lived.	19.2	15.4	35.9	20.5	9	6
**Internal reasons**						
I had been worried about what others would think about me.	7.3	12.2	34.1	37.8	8.5	2
I was too proud to ask for help.	10.1	12.7	22.8	34.2	20.3	5
I felt unable to discuss my problems with others.	6.3	16.5	27.8	35.4	13.9	5
I did not want people to think of me as an addict or mentally disordered.	11.1	21	22.2	25.9	19.8	3
I was afraid of feeling like a failure if I could not get away from gambling despite help.	16.2	13.8	30	23.8	16.2	4
**Attitude**						
I thought I could handle it on my own.	6	13.3	20.5	28.9	31.3	1
I did not want to admit to myself that I needed help.	8.8	10	38.8	31.2	11.2	4
I felt that gambling was not a big problem in my life.	7.3	11	31.7	36.6	13.4	2
**Support**						
My family and friends did not support me enough in seeking help.	15.6	19.5	29.9	27.3	7.8	7

*n* = 84. 1 = not at all; 2 = to a small extent; 3 = to a moderate extent; 4 = to a large extent; 5 = to a great extent/completely.

For the ease of reading, the statements have been grouped together. Participants were considered to have endorsed a statement if they responded with either 3 “to a moderate extent”; 4 “to a large extent” or 5 “to a great extent/completely.” The statement “*My family and friends did not support me enough in seeking help*,” which was assigned to the dimension of “support,” has the lowest level of endorsement (65%). The two statements that were most frequently endorsed (81.7%) were, “*I thought that treatment would cost me too much time and energy*” assigned to the “Treatment related” dimension of and, “*I felt that gambling was not a big problem in my life*,” assigned to the “attitude” dimension. It is striking that all statements in the “attitude” dimension show endorsement ratings above 80% (mean: 81.2%), while the mean value of the dimension of “internal reason” is 74.5% and the mean value of the “treatment related” dimension is 72%. Of course, it is important for the interpretation that only trends are shown, but nevertheless, the cautious conclusion can be drawn that the gambler’s attitude is a bigger barrier than treatment-related reasons.

## Discussion

The study is part of a broader research project examining the influence of exclusion on casino gamblers in terms of various indicators, such as the motivation to seek help. In addition to the influence of exclusion on help-seeking, the present study examines other factors that encourage gamblers with at-risk gambling behavior or GD to request help. Regarding exclusion, a relationship can be confirmed between being excluded and the subsequent use of help services. Six months after the baseline survey, it can be observed that the share of excluded gamblers among those seeking help is significantly higher. The relationship between exclusion and help-seeking is thus in line with previous findings (24, 25). However, the participants in this sub-group can also be assumed to have a more pronounced burden of harm and therefore other factors must also be considered. It is likely that one’s own recognition of gambling problems is a crucial and this was certainly identified as a significant factor for help-seeking within the present study.

Previous findings indicate that problem gamblers will seek help only when gambling problems become severe (9). This finding was also upheld by the analysis. The proportion of help-seekers with a SOGS-R score ≥ 5 is significantly higher than among gamblers who have “some problems” according to SOGS-R. It is important to note, however, that a number of factors are involved in help-seeking factors and that this does not necessarily mean gamblers have “hit rock bottom.” (11). The survey also confirmed that having debts is a driver for seeking help. Overall, the results are plausible and in line with previous studies (8–11, 14, 16, 48). However, current research suggests that demographic variables, including age, gender, geographic location and cultural background may also impact treatment-seeking behavior (8). At this point, the results of the present study differ from the established research, as an influence of age and gender could not be observed. Similarly, the form of gambling does not appear to influence the motivation to seek help. The exception being for gamblers at land-based casinos, where the percentage of people who do seek help was significantly lower. However, previous research suggests that some forms of gambling (e.g., electronic games machines, casino games and some types of sports betting) are more closely associated with GD than other forms (e.g., lotteries) (49), which implies a relationship not found in the present study. The state of research regarding different levels of potential risk for different gambling products is plausible. The deviation in the study at hand can presumably be explained by the fact that the survey was conducted in the same setting, and thus under comparable circumstances. The participants are primarily casino gamblers, even if some participate in other forms of gambling. This is a possible reason why there is no significant difference regarding the type of gambling reported, in relation to help-seeking behavior.

A further research question was to find out what type of gambling-specific help services gamblers used in the past 6 months. Twenty-three individuals, (10.7% of the sample who participated in the second wave of the survey) reported using help services. The most widely used forms of support were specialized addiction centers, which by their nature included psychotherapists and psychiatrists (65.2%), followed by self-help groups (34.8%) and remote counseling services (17.4%). 17.4% of respondents reported having sought support from significant others. The results suggest that self-help services should receive more attention in the research literature, and also from public health practitioners. To date, however, there has been relatively little research on the benefits of self-help groups, even though it can be assumed that there is a greater willingness to use this type of professional service, as opportunities to meet other affected people can reduce the feeling of stigmatization (21). Mutual support groups, as a form of self-help group were recognized a few years ago in Swedish study. Particularly, the authors demonstrate how participants at the group meetings give and receive support at different stages on the path to recovery and at different levels (20). Within the present study, it is notable that the land-based services offered by addiction counseling centers and self-help groups are used more than their remote counterparts. However, research suggests that the option of a web-based self-help program or self-management tools could potentially reach those gamblers who are hesitant to approach specialized addiction centers and help them to reduce or stop their problem gambling (50). It is therefore important that relevant programs are more actively promoted.

In the context of the overarching research question, it is important to explore why individuals with at-risk gambling behavior or GD have not sought help, to date. This question has already been researched in depth in other studies. For example, one review found that the most frequently cited barriers were the wish to handle problems by oneself; shame/embarrassment/stigma; unwillingness to admit there is a problem; and issues with the treatment, itself (6). Further results indicated that seeking professional help is predominantly crisis-driven rather than being motivated by a gradual recognition of problematic behavior (9). Shame, denial and pride seems thus to be the most significant barriers to change rather than a lack of knowledge, or dislike of treatment agencies (9, 51). Against this background, the question of barriers to help-seeking was also addressed in the present study. Gamblers who had not used help in the last 6 months could choose from the response categories “no, because I do not have problems” and “no, not yet.” The latter (*n* = 84) were asked about the specific barriers. Overall, the responses of the gamblers tended to indicate that attitudes towards help-seeking (e.g., denial) pose a greater barrier than, for example, reasons related to gambling-specific help services, although the latter also have a high level of influence. However, it is important to take an in-depth look at the individuals who indicated that they do not have any gambling-related problems and therefore did not seek help. In this group 90.9% of the 66 individuals can be considered as gamblers with some problems. According to SOGS-R, 9.1% shows a probable pathological gambling behavior. The finding that “denial” is an important barrier can thus be confirmed in an indirect manner. The fact that the barrier “denial” is a weighty factor can also be seen in another context. Altogether, five individuals with a SOGS-R score of 0 stated that they had sought help. This seems somewhat contradictory and can be explained by the fact that the denial of respective problems is part of GD. Hence, the reason why the gamblers nevertheless sought help cannot be conclusively plausibly based on the available data.

In general, the effects of stigma on people experiencing gambling problems are apparent through the low rates of problem disclosure and treatment-seeking that are currently observed (22). Thus, strategies designed to raise problem awareness, destigmatize problem gambling and normalize treatment-seeking behavior should be prioritized (51). From a public health perspective, a mix of public education campaigns, awareness campaigns, advertising campaigns, and early intervention in casinos is needed (52). Better public understanding could encourage greater awareness of the nature of GD as a behavioral addiction rather than a character defect (53). In addition, public education campaigns could aim to reduce the stigma associated with problem gambling, as well as being harmed by someone else’s gambling, which could help to improve the uptake of help services (54). People with GD should be encouraged to view help-seeking as responsible step and not a sign of weakness. The act of seeking help must be seen as an act of strength that should be applauded. In addition, advertising for specialized problem gambling services should be designed to emphasize the fact that there are others who experience gambling problems and that problem gambling can be treated and overcome (52). Early intervention in casinos, as mentioned above, is also an important preventive component. Moreover, the processes in Swiss casinos stipulate that gamblers who are excluded must be made aware of the services offered by the addiction centers (55). In addition, each casino has a specialist addiction center as a partner and is obliged to ensure that the interface with the addiction support system is well designed (26). The above strategy has the potential to increase the rate of gamblers seeking help. The more gamblers with GD that seek specialist assistance, and the earlier that help is sought, the greater the opportunity to reduce, resolve or prevent the associated harms (51). However, in the context of this study, only some of the excluded gamblers stated that they had been made aware of the counseling services by casino employees. Meanwhile, it is important to bear in mind that appropriately addressing a person with GD by casino staff is a very demanding task and requires tailored training. Swiss legislation obliges casinos to have casino staff who are regularly trained (26). If positive change toward higher rates of support for at-risk gamblers is to be achieved, it is necessary to understand, as part of this training, the barriers that prevent staff from making proactive interventions when faced with identified gamblers (56).

## Limitations

Several limitations that may have influenced the results should be identified. The data collected were self-reported and may be subject to recall bias, social desirability bias, and other distortions. Furthermore, since hardly any gamblers with a migrant background participated in the study, a selection bias can be assumed. Due to this low level of representation, it was not possible to analyze factors relating to migration status.

Another important limitation stems from the sample design. The study at hand is part of a broader research project investigating the influence of exclusion on various indicators, such as the motivation to seek help. For this purpose, excluded and non-excluded gamblers were recruited. Given this research design, excluded gamblers are overrepresented in the present study, and therefore the proportion of gamblers with problem gambling behavior.

Due to the small sample size, some analyses could not be performed that might have revealed important findings. For example, whether differences can be observed in the various help services used with regard to the user’s demographic characteristics (e.g., age, gender, severity of the GD). Overall, it would have been important to trace the trajectories of those gamblers who sought help. Due to the number of cases, only individual experiences could have been described, but no general statements could have been made, which is why this procedure was not undertaken.

Finally, it is likely that the Covid pandemic influenced the results. During the survey period, land-based casinos were closed from March to June 2020 and from November 2020 to January 2021. This was taken into account by asking gamblers to consider only the time outside the lockdown in terms of game participation. However, it is entirely possible that the ongoing context of the pandemic also influenced gambling behaviors during the period that the study was carried out.

## Conclusion

Overall, the findings can be taken to demonstrate that exclusion appears to be an effective harm reduction measure. The present study confirms findings from previous studies that this measure not only prevents individuals from generating further financial losses but may also motivate gamblers to seek help from sources of support. Other factors identified in the study that encourage gamblers to seek help include debt, recognition of their own gambling problems, and the severity of gambling-related problems. Meanwhile, the share of gamblers who seek help remains low. It is therefore the task of public health practitioners to increase this proportion through the use of targeted measures.

Self-help groups seem to be a viable option to support gamblers with GD. Corresponding offers should be promoted more strongly, both by public health practitioners and by gambling operators. Overall, it is surprising that this service has not been the focus of more research, to date. Moreover, although the addiction help system now provides numerous and elaborate distance-based services (e.g., helplines), the gamblers questioned in the present study preferred face-to-face services. This suggests that corresponding services need to be better promoted and perhaps also optimized. Given that remote services are a promising option for people with GD who do not want to visit an addiction counseling center due to shame or fear of stigmatization, it would be worthwhile to conduct further research to develop distance-based services. Furthermore, it is important that trained casino employees inform on and promote the help available.

From a public health perspective, an overarching strategy is required to ensure needs-oriented help. This should be a mixture of different preventive measures. The interface between the Addiction Help System and the casino must be considered. To rely on a basis for needs-based planning, regular monitoring for GD is required, which reflects not only the burden of gambling-related harm but also figures that demonstrate the uptake of services, in a differentiated manner.

## Data availability statement

The raw data supporting the conclusions of this article will be made available by the authors, without undue reservation.

## Ethics statement

The studies involving human participants were reviewed and approved by The Swiss Ethical Authority decided that the project did not require formal ethical approval since it does not involve research on human diseases or the structure and function of the human organism (file number Req-2019-00060). The patients/participants provided their written informed consent to participate in this study.

## Author contributions

SL and JS: conceptualization. SL: funding acquisition, project administration. JS: methodology. JM: data curation. JS and HW: formal analysis. The first draft of the manuscript was written by SL and EJ. All authors contributed to the article and approved the submitted version.

## Funding

This research was funded by the Swiss National Science Foundation (SNSF), grant number 10001A_178811. Open access funding by Lucerne University of Applied Sciences and Arts.

## Conflict of interest

The authors declare that the research was conducted in the absence of any commercial or financial relationships that could be construed as a potential conflict of interest.

## Publisher’s note

All claims expressed in this article are solely those of the authors and do not necessarily represent those of their affiliated organizations, or those of the publisher, the editors and the reviewers. Any product that may be evaluated in this article, or claim that may be made by its manufacturer, is not guaranteed or endorsed by the publisher.
