# Corrigendum: Motivating factors and barriers to help-seeking for casino gamblers: results from a survey in Swiss casinos

**DOI:** 10.3389/fpsyt.2023.1244080

**Published:** 2023-08-08

**Authors:** Suzanne Lischer, Jürg Schwarz, Hannes Wallimann, Emilien Jeannot, Jacqueline Mathys

**Affiliations:** ^1^Lucerne University of Applied Sciences and Arts, Lucerne, Switzerland; ^2^Centre Du Jeu Excessif, Addiction Medicine, Lausanne University Hospital, Lausanne, Switzerland; ^3^Institute of Global Health, Faculty of Medicine, Chemin de Mines, Geneva, Switzerland

**Keywords:** problem gambling, help-seeking, casino, exclusion, public health, disordered gambling, gambling-specific help service

In the published article, percentages were misrepresented in Table 4, “Use of gambling-specific help services (SOGS-R ≥ 1, *n* = 23, multiple choice)”.

The adjusted percentages are shown below.

**Table 4:** Use of gambling-specific help services (SOGS-R ≥ 1, n = 23, multiple choice).

Self-help groups	34.8% (8)
Online self-help groups	26.1% (6)
Counseling services, psychiatrist or psychotherapist	65.2% (15)
Online counseling services	17.4% (4)
Debt counseling	8.7% (2)
Addiction treatment inpatient services	4.3% (1)
Significant others	17.4% (4)
Other	4.3% (1)

The incorrectly presented percentages in Table 4 were reproduced in the text in two places.

A correction was made in the **Results**, *Type of gambling-specific help service*, second Paragraph. These sentences previously stated:

“Outpatient addiction counseling services are the most used form of gambling-specific help service (39.5% uptake). Self-help groups are the second most frequently used help service (21.1%).

The corrected sentences appear below:

“Outpatient addiction counseling services are the most used form of gambling-specific help service (65.2% uptake). Self-help groups are the second most frequently used help service (34.8%).

Another correction was made in the **Discussion**, third Paragraph. These sentences previously stated:

“Twenty three individuals, (10.7% of the sample who participated in the second wave of the survey) reported using help services. The most widely used forms of support were specialized addiction centers, which by their nature included psychotherapists and psychiatrists (39.5%), followed by self-help groups (21.1%) and remote counseling services (10.5%). 10.5% of respondents reported having sought support from significant others.”

The corrected sentences appear below:

“Twenty-three individuals, (10.7% of the sample who participated in the second wave of the survey) reported using help services. The most widely used forms of support were specialized addiction centers, which by their nature included psychotherapists and psychiatrists (65.2%), followed by self-help groups (34.8%) and remote counseling services (17.4%). 17.4% of respondents reported having sought support from significant others.”

The authors apologize for this error and state that this does not change the scientific conclusions of the article in any way. The original article has been updated.

