# Supplementary material for: Motivating factors and barriers to help-seeking for casino gamblers: results from a survey in Swiss casinos
Source: Front Psychiatry. 2023 May 25;14:1128291. doi: 10.3389/fpsyt.2023.1128291 (PMC10249729; doi:10.3389/fpsyt.2023.1128291)
Supplement: Supplementary file 1 [file Data_Sheet_1.PDF]

Lucerne University of
Applied Sciences and Arts
HOCHSCHULE
LUZERN
FH Zentralschweiz

FNSNF
Schweizerischer Nationalfonds
zur Förderung der wissenschaftlichen Forschung

Glücksspielstudie

Fragebogen zur Spielsperre als Massnahme des Spielerschutzes

Jacqueline Mathys, Suzanne Lischer,
Angela Steffen, Jürg Schwarz

Luzern | 12. 08. 2019

Fragebogen Teil 1

Hochschule Luzern
Werftestrasse 1
Postfach 2945
CH-6002 Luzern

Suzanne.Lischer@hslu.ch

Ausfüllungsanweisungen
Je nach Ihrer Situation müssen Sie nicht alle Fragen beantworten. Wir haben dies mit einem Pfeil und der Fragenummer gekennzeichnet, zu der Sie springen können. Beispielsweise bei Frage A.3, wenn Sie hier Schweiz angegeben haben, können Sie mit der Frage A.5 weiterfahren und die Frage A.4 auslassen.

Um aussagekräftige Ergebnisse zu erhalten, ist es wichtig, dass Sie die Fragen sorgfältig durchlesen und wenn möglich alle Fragen beantworten. Wenn Sie eine Frage nicht beantworten möchten, können Sie die Option «keine Angabe» wählen.

A. Angaben zu Ihrer Person

A.1 Bitte geben Sie Ihr Geschlecht an: ☐ Weiblich ☐ Männlich ☐ Divers ☐ Keine Angabe

A.2 In welchem Jahr sind Sie geboren? ______ (Jahr, z. B. 1966)

A.3 In welchem Land sind Sie geboren? ______ → Falls Schweiz, weiter mit **Frage A.5**

A.4 Seit wann leben Sie in der Schweiz? ______ (Jahr, z. B. 1983)

A.5 Welche Staatsbürgerschaft(en) haben Sie? *Falls Sie mehrere haben, geben Sie bitte die für Sie wichtigsten beiden an.* ______

A.6 Welche Staatsbürgerschaft hat/hatte Ihre Mutter? ______ Ihr Vater? ______

A.7 Welchen rechtlichen Familienstand haben Sie?
- ☐ Ledig
- ☐ Verheiratet / In einer registrierten Partnerschaft
- ☐ Geschieden / Aufgelöste registrierte Partnerschaft
- ☐ In einer Beziehung
- ☐ Verwitwet
- ☐ Keine Angabe

A.8 Leben Sie mit Erwachsenen zusammen?
- ☐ Nein
- ☐ Ja, in einer Wohngemeinschaft
- ☐ Anderes
- ☐ Ja, mit dem/der Ehe- oder Lebenspartner/in
- ☐ Ja, mit den Eltern, einem Elternteil oder Verwandten
- ☐ Keine Angabe

A.9 Haben Sie unterstützungspflichtige Kinder?
- ☐ Ja ___ (Anzahl), in meinem Haushalt lebend.
- ☐ Nein
- ☐ Keine Angabe
- ☐ Ja ___ (Anzahl), nicht in meinem Haushalt lebend.

A.10 Welches ist Ihre höchste abgeschlossene Ausbildung?
- ☐ Kein abgeschlossener obligatorischer Schulabschluss
- ☐ Berufslehre oder Vollzeit-Berufsschule, Berufsmatura
- ☐ Universität, Hochschule, Fachhochschule
- ☐ Obligatorische Schule
- ☐ Diplom- oder Fachmittelschule, Maturität, Lehrerseminar
- ☐ Eine andere Ausbildung
- ☐ Anlehre, 1 Jahr Handelsschule, Haushaltslehrjahr, 10. Schuljahr
- ☐ Technikum, Höhere Fachschule (HTL/HWV), Höherer Fachausweis
- ☐ Keine Angabe

A.11 Wie hoch ist Ihr persönliches monatliches Nettoeinkommen?
- ☐ Weniger als CHF 3’000
- ☐ CHF 7’001 – 9’000
- ☐ Mehr als CHF 13'000
- ☐ CHF 3'000 – 5'000
- ☐ CHF 9’001 – 11’000
- ☐ Keine Angabe
- ☐ CHF 5’001 – 7'000
- ☐ CHF 11'001 – 13’000

A.12 Welche Form der Erwerbstätigkeit üben Sie zurzeit hauptsächlich aus?
- ☐ Selbstständig
- ☐ Hausfrau/Hausmann
- ☐ Student/in
- ☐ Angestellt
- ☐ Sozialhilfe-Bezüger/in
- ☐ Sonstiges
- ☐ Im Ruhestand (AHV)
- ☐ ALV-Bezüger/in
- ☐ Keine Angabe
- ☐ IV-Bezüger/in
- ☐ In Ausbildung, Praktikum

B. Fragen zum Spielverhalten

Die weiteren Fragen beziehen sich auf Ihr Spielverhalten. Unter dem Sammelbegriff Glücksspiele sind alle Spiele und Wetten mit Geldeinsatz zu verstehen.

B.1 Bitte geben Sie an, wie gut die folgenden Aussagen auf Sie zutreffen:	Trifft gar nicht zu	Trifft wenig zu	Trifft etwas zu	Trifft ziemlich zu	Trifft voll und ganz zu	Keine Angabe
Ich spiele/spielte Glücksspiele …						
… weil es Spass macht	☐	☐	☐	☐	☐	☐
… aus Gewohnheit	☐	☐	☐	☐	☐	☐
… wegen des Nervenkitzels	☐	☐	☐	☐	☐	☐
… weil meine Freund/innen auch spielen	☐	☐	☐	☐	☐	☐
… wenn mir langweilig ist	☐	☐	☐	☐	☐	☐
… weil ich dadurch alles um mich herum vergessen kann	☐	☐	☐	☐	☐	☐
… weil ich damit Geld verdienen will	☐	☐	☐	☐	☐	☐
… weil man so leichter unter Leute kommt	☐	☐	☐	☐	☐	☐
… wenn ich gestresst oder deprimiert bin oder Sorgen habe	☐	☐	☐	☐	☐	☐
… wegen der Glücks- und Erfolgsgefühle	☐	☐	☐	☐	☐	☐
… wegen dem Prestige und/oder dem Ambiente	☐	☐	☐	☐	☐	☐

B.2 Nachfolgend finden Sie eine Liste zu **Glücksspielen in der Schweiz,** zu **Glücksspielen im Ausland** und zu **Glücksspielen im Internet**.

Bitte geben Sie an, wie oft und wie lange Sie in den **letzten 6 Monaten** daran teilgenommen haben. Gesperrte Spieler/innen beziehen sich bitte auf die **6 Monate vor der Spielsperre**.

	Wie oft haben Sie teilgenommen?							Wie viele Stunden haben Sie jeweils damit verbracht, wenn Sie spielten?
**Glücksspiele in der Schweiz (offline)**	nie	Bis 1 x im Monat	1 – 3 x im Monat	1 – 2 x pro Woche	3 – 4 x pro Woche	5 – 6 x pro Woche	täglich	Angabe in h
Roulette, Black Jack, andere Tischspiele in Casinos (ohne Poker)	☐	☐	☐	☐	☐	☐	☐	____
Glücksspielautomaten (Slots) in Casinos	☐	☐	☐	☐	☐	☐	☐	____
Poker in Casinos	☐	☐	☐	☐	☐	☐	☐	____
Pokerturniere (kleine Pokerturniere ausserhalb von Casinos)	☐	☐	☐	☐	☐	☐	☐	____
Poker privat (Freund/innen und Familie)	☐	☐	☐	☐	☐	☐	☐	____
Glücksspiele in den «Hinterzimmern» von Clubs, Bars und Vereinslokalen	☐	☐	☐	☐	☐	☐	☐	____
Poker in den «Hinterzimmern» von Clubs, Bars und Vereinslokalen	☐	☐	☐	☐	☐	☐	☐	____

	Wie oft haben Sie teilgenommen?							Wie viele Stunden haben Sie jeweils damit verbracht, wenn Sie spielten?
	nie	Bis 1 x im Monat	1 – 3 x im Monat	1 – 2 x pro Woche	3 – 4 x pro Woche	5 – 6 x pro Woche	täglich	Angabe in h
Sportwetten (Fussball, etc.)	☐	☐	☐	☐	☐	☐	☐	
Sportwetten in den «Hinterzimmern» von Clubs, Bars und Vereinslokalen	☐	☐	☐	☐	☐	☐	☐	
Pferdewetten (Swisslos, LoRo)	☐	☐	☐	☐	☐	☐	☐	
Tactilo (elektronische Lotterien)	☐	☐	☐	☐	☐	☐	☐	
Lotto, Lose am Kiosk (Swisslos, LoRo)	☐	☐	☐	☐	☐	☐	☐	
Sonstige Glücksspiele	☐	☐	☐	☐	☐	☐	☐	
**Glücksspiele im Ausland (offline)**								
Glücksspiele in Casinos im Ausland	☐	☐	☐	☐	☐	☐	☐	
Spielhallen im Ausland	☐	☐	☐	☐	☐	☐	☐	
Sonstige Glücksspiele im Ausland	☐	☐	☐	☐	☐	☐	☐	
**Glücksspiele im Internet**								
Glücksspiele auf Internetseiten von Schweizer Casinoanbietern	☐	☐	☐	☐	☐	☐	☐	
Glückspiele auf Internetseiten von anderen Anbietern	☐	☐	☐	☐	☐	☐	☐	
Poker auf Internetseiten von Schweizer Casinoanbietern	☐	☐	☐	☐	☐	☐	☐	
Poker auf Internetseiten von anderen Anbietern	☐	☐	☐	☐	☐	☐	☐	
Sportwetten auf Internetseiten von Schweizer Anbietern (Swisslos, LoRo)	☐	☐	☐	☐	☐	☐	☐	
Sportwetten auf Internetseiten von anderen Anbietern	☐	☐	☐	☐	☐	☐	☐	
Lotto auf Internetseiten von Schweizer Anbietern (Swisslos, LoRo)	☐	☐	☐	☐	☐	☐	☐	
«Spekulationen» an der Börse oder auf dem Optionsmarkt	☐	☐	☐	☐	☐	☐	☐	
Sonstige Glücksspiele im Internet	☐	☐	☐	☐	☐	☐	☐	

B.3 Welches der von Ihnen angegebenen Glücksspiele hatte in den letzten 6 Monaten die grösste Bedeutung für Sie?

B.4 Wussten Sie, dass Schweizer Casinos seit dem 01.01.2019 auf ihren eigenen Webseiten legale Online-Glücksspiele anbieten dürfen?

☐ Ja ↓

☐ Nein → weiter mit **Frage B.8**

☐ Keine Angabe → weiter mit **Frage B.8**

B.5 Nutzen Sie dieses Angebot?

- ☐ Ja ↓
- ☐ Nein → `weiter mit` **`Frage B.7`**
- ☐ Keine Angabe → `weiter mit` **`Frage B.7`**

B.6 Aus welchem Grund nutzen Sie das neue Online-Angebot der Schweizer Casinos?

- ☐ Weil es legal ist
- ☐ Habe höheres Vertrauen in Schweizer Casinos
- ☐ Attraktiveres Angebot
- ☐ Weiss nicht
- ☐ Keine Angabe
- ☐ Anderer Grund: ____

B.7 Aus welchem Grund nutzen Sie das neue Online-Angebot der Schweizer Casinos nicht?

- ☐ Ich mag generell keine Online-Spiele
- ☐ Ich ziehe das Angebot von Anbietern aus dem Ausland vor
- ☐ Weiss nicht
- ☐ Keine Angabe
- ☐ Anderer Grund: ____

B.8 Haben Sie beim Spielen jemals einen hohen Gewinn erzielt? `(offline oder online)`

- ☐ Nein
- ☐ Ja, nämlich: ____ CHF
- ☐ Keine Angabe

B.9 Welchen Geldbetrag haben Sie in den letzten 6 Monaten durchschnittlich im Monat für Glücksspiele ausgegeben?

- ☐ Weniger als CHF 10
- ☐ CHF 10 – 99
- ☐ CHF 100 – 299
- ☐ CHF 300 – 499
- ☐ CHF 500 – 999
- ☐ CHF 1000 – 2'499
- ☐ CHF 2500 – 9'999
- ☐ CHF 10'000 und mehr
- ☐ Weiss nicht
- ☐ Keine Angabe

B.10 Haben Sie eine Einsatzlimite für Glücksspiele?

- ☐ Ja ↓
- ☐ Nein → `weiter mit` **`Frage B.12`**
- ☐ Keine Angabe → `weiter mit` **`Frage B.12`**

B.11 Fällt es Ihnen oft schwer, sich an diese Limite zu halten?

- ☐ Nie
- ☐ Selten
- ☐ Manchmal
- ☐ Oft
- ☐ Immer
- ☐ Keine Angabe

B.12 Haben Sie in den vergangenen 6 Monaten von jemandem Geld geliehen und dieses aufgrund Ihres Spielens nicht zurückgezahlt?

- ☐ Ja
- ☐ Nein

B.13 Wenn Sie sich Geld zum Spielen oder für die **Rückzahlung** von Spielschulden geliehen haben, geben Sie bitte an, von wo oder wem Sie sich das Geld geliehen haben. (Mehrfachnennungen möglich)

- ☐ Ich habe mir kein Geld geliehen
- ☐ Von Banken, Finanz- oder Kreditinstitutionen
- ☐ Vom Verkauf von persönlichem oder familiärem Vermögen/Eigentum
- ☐ Vom Haushaltsgeld
- ☐ Über Kreditkarten
- ☐ Überziehung des Kontos (bzw. Ausstellung ungedeckter Schecks)
- ☐ Vom/von der Ehe-/Lebenspartner/in
- ☐ Von «Kredithaien»
- ☐ Von Freund/innen
- ☐ Von anderen Verwandten (auch angeheiratete)
- ☐ Vom Verkauf von Aktien, Wertpapieren oder anderen Anlagen
- ☐ Ich habe/hatte einen Kredit bei einem/r Buchmacher/in

B.14 Haben Sie Schulden aufgrund des Glücksspiels?

- ☐ Nein
- ☐ Weiss nicht
- ☐ Keine Angabe
- ☐ Ja, insgesamt ca.: ____ CHF

B.15 Haben Sie illegale Handlungen wie Fälschung, Betrug, Diebstahl oder Unterschlagung begangen, um das Glücksspiel zu finanzieren oder Spielschulden zu begleichen?

- ☐ Ja
- ☐ Nein
- ☐ Keine Angabe

Bei manchen Personen können Probleme aufgrund des Glücksspielens auftreten.
Wir würden gerne wissen, wie das bei Ihnen ist.

B.16 Haben Sie den Eindruck, dass Sie in den vergangenen 6 Monaten ein Problem mit Glücksspielen oder Geldwetten gehabt haben?

- ☐ Ja ↓
- ☐ Nein → `weiter mit` **`Frage B.18`**

B.17 Wie lange besteht dieses Problem schon?

- ☐ ______ Jahre
- ☐ Weiss nicht
- ☐ Keine Angabe

B.18 Wenn Sie in den vergangenen 6 Monaten gespielt haben, wie häufig versuchten Sie später durch erneutes Spielen Geldverluste zurück zu gewinnen?

- ☐ Niemals in den vergangenen 6 Monaten
- ☐ Bei Geldverlusten meistens
- ☐ Manchmal (weniger als die Hälfte der Male an denen ich Geld verloren habe)
- ☐ Immer nach Geldverlusten

B.19 Haben Sie in den vergangenen 6 Monaten behauptet, dass Sie beim Spielen Geld gewonnen haben, aber in Wirklichkeit verloren hatten?

- ☐ Niemals in den vergangenen 6 Monaten
- ☐ Ja, manchmal (weniger als die Hälfte der Male, bei denen ich verloren hatte)
- ☐ Ja, meistens

Die folgenden Fragen beziehen sich auf die letzten 6 Monate.

	Ja	Nein
B.20 Haben Sie mehr gespielt als Sie beabsichtigt hatten?	☐	☐
B.21 Haben andere Menschen Ihr Wettverhalten kritisiert oder Ihnen gesagt, Sie hätten ein Spielproblem, unabhängig davon, ob Sie dem zustimmen oder nicht?	☐	☐
B.22 Haben Sie sich schuldig gefühlt in Bezug auf die Art wie Sie in dieser Zeit gespielt haben oder was passierte, wenn Sie spielten?	☐	☐
B.23 Hatten Sie den Wunsch, mit dem Spielen oder Wetten aufzuhören, fühlten sich aber gleichzeitig unfähig dazu?	☐	☐
B.24 Haben Sie Spielbelege, Lotterietickets, Spielgeld, Schuldscheine oder andere Anzeichen für Wetten oder Spielen vor Ihrem/Ihrer Ehe-/Lebenspartner/in, Ihren Kindern oder anderen wichtigen Personen in Ihrem Leben versteckt?	☐	☐
B.25 Haben Sie bei der Arbeit oder während des Schulunterrichtes gefehlt, um zu spielen?	☐	☐
B.26 Haben Sie sich mit Menschen, mit denen Sie zusammenleben über Ihren Umgang mit Geld gestritten?	☐	☐
Wenn ja, bezog sich der Streit um Geld auf Ihr Spielverhalten?	☐	☐

B.27 Gibt es in Ihrem sozialen Umfeld Personen, die Ihrer Meinung nach ein **Spielproblem** haben oder hatten?

- ☐ Ja ↓
- ☐ Nein → `weiter mit` **`Frage C.1`**
- ☐ Weiss nicht → `weiter mit` **`Frage C.1`**
- ☐ Keine Angabe → `weiter mit` **`Frage C.1`**

B.28 Markieren Sie, welche der folgenden Personen in ihrem Leben ein Spielproblem haben oder hatten.

- ☐ Ehe-/Partner/in
- ☐ Vater
- ☐ Kinder
- ☐ Freund/innen
- ☐ Mutter
- ☐ Geschwister
- ☐ Sonstige Verwandte, die Ihnen nahestehen

C. Fragen zur Lebensqualität

C.1 Die folgenden Aussagen betreffen Ihr Wohlbefinden in den letzten 2 Wochen. Bitte wählen Sie bei jeder Aussage die Antwort, die Ihrer Meinung nach am besten beschreibt, wie Sie sich in den letzten zwei Wochen gefühlt haben.

In den letzten 2 Wochen ...	Die ganze Zeit	Meistens	Etwas mehr als die Hälfte der Zeit	Etwas weniger als die Hälfte der Zeit	Ab und zu	Zu keinem Zeitpunkt
... war ich froh und guter Laune.	☐	☐	☐	☐	☐	☐
... habe ich mich ruhig und entspannt gefühlt.	☐	☐	☐	☐	☐	☐
... habe ich mich energisch und aktiv gefühlt.	☐	☐	☐	☐	☐	☐
... habe ich mich beim Aufwachen frisch und ausgeruht gefühlt.	☐	☐	☐	☐	☐	☐
... war mein Alltag voller Dinge, die mich interessieren.	☐	☐	☐	☐	☐	☐

C.2 Wie gut beschreiben die folgenden Aussagen Ihre Handlungen und Verhaltensweisen?

	Trifft gar nicht zu	Trifft wenig zu	Trifft etwas zu	Trifft ziemlich zu	Trifft voll und ganz zu	Keine Angabe
In schwierigen Situationen kann ich mich auf meine Fähigkeiten verlassen.	☐	☐	☐	☐	☐	☐
Die meisten Probleme kann ich aus eigener Kraft gut meistern.	☐	☐	☐	☐	☐	☐
Auch anstrengende und komplizierte Aufgaben kann ich in der Regel gut lösen.	☐	☐	☐	☐	☐	☐

C.3 Wie oft fühlten Sie sich im Verlauf der letzten 2 Wochen durch die folgenden Beschwerden beeinträchtigt?

	Überhaupt nicht	An einzelnen Tagen	An mehr als der Hälfte der Tage	Beinahe jeden Tag	Keine Angabe
Wenig Interesse oder Freude an Ihren Tätigkeiten	☐	☐	☐	☐	☐
Niedergeschlagenheit, Schwermut oder Hoffnungslosigkeit	☐	☐	☐	☐	☐
Nervosität, Ängstlichkeit oder Anspannung	☐	☐	☐	☐	☐
Nicht in der Lage sein, Sorgen zu stoppen oder zu kontrollieren	☐	☐	☐	☐	☐

C.4 Nun geht es um Ihre allgemeine Lebenszufriedenheit. Wie zufrieden sind Sie gegenwärtig, alles in allem, mit Ihrem Leben? *(0 bedeutet gar nicht zufrieden und 10 bedeutet vollumfänglich zufrieden.)*

gar nicht zufrieden — vollumfänglich zufrieden

0	1	2	3	4	5	6	7	8	9	10
☐	☐	☐	☐	☐	☐	☐	☐	☐	☐	☐

C.5 Bitte geben Sie an wie zufrieden Sie gegenwärtig mit folgenden Lebensbereichen sind: *(0 bedeutet gar nicht zufrieden und 10 bedeutet vollumfänglich zufrieden.)*

Wie zufrieden sind Sie mit ...	0 (gar nicht zufrieden)	1	2	3	4	5	6	7	8	9	10 (vollumfänglich zufrieden)
... Ihrer finanziellen Situation?	☐	☐	☐	☐	☐	☐	☐	☐	☐	☐	☐
... Ihren persönlichen Beziehungen?	☐	☐	☐	☐	☐	☐	☐	☐	☐	☐	☐
... Ihrer Freizeit?	☐	☐	☐	☐	☐	☐	☐	☐	☐	☐	☐
... Ihrer Wohnsituation?	☐	☐	☐	☐	☐	☐	☐	☐	☐	☐	☐
... Ihrer Gesundheit im Allgemeinen?	☐	☐	☐	☐	☐	☐	☐	☐	☐	☐	☐
... Ihrer beruflichen Situation?	☐	☐	☐	☐	☐	☐	☐	☐	☐	☐	☐

D. Fragen zur Spielsperre

D.1 Sind Sie aktuell für das Glücksspiel in einem Schweizer Casino gesperrt? **Pflichtfrage!** Diese Frage ist von grosser Wichtigkeit. Bitte beantworten.

☐ Ja ↓ ☐ Nein → weiter mit **Frage D.14**

D.2 Wann wurde die Spielsperre ausgesprochen? **Pflichtfrage!**

__________ Datum (z. B. 20.2.2019)

D.3 Welches Angebot war der Grund für die Spielsperre? **Pflichtfrage!**

☐ Glücksspiel im Casino ☐ Glücksspiel im Internet ☐ Swisslos, LoRo

D.4 Um welche Art der Spielsperre handelt es sich? **Pflichtfrage!**

☐ Angeordnete Spielsperre ↓

☐ Freiwillige Spielsperre → weiter mit **Frage D.9**

Für angeordnete Spielsperren:

D.5 Wurden Sie aus Ihrer Sicht zu früh, rechtzeitig oder zu spät gesperrt?

☐ Zu früh ☐ Rechtzeitig ☐ Zu spät ☐ Keine Angabe

D.6 Aus welchem Grund wurden Sie angeordnet gesperrt?

☐ Keinen Finanzausweis erbracht ☐ Meldung Dritter ☐ Keine Angabe

☐ Meldung Sozialbehörde, Fachstelle ☐ Spielsperre wegen Falschspiel / Täuschung ☐ Anderer Grund: __________

D.7 War die Verhängung der Spielsperre aus Ihrer Sicht gerechtfertigt?

☐ Ja ☐ Nein ☐ Keine Angabe

D.8 Aus welchem Hauptgrund haben Sie keine freiwillige Spielsperre beantragt? (Bitte geben Sie nur den Hauptgrund an)

- ☐ Es gibt keinen Grund für eine Spielsperre.
- ☐ Ich wollte weiterhin an Glücksspielen teilnehmen
- ☐ Ich wollte zuerst das verlorene Geld zurückgewinnen
- ☐ Eine freiwillige Spielsperre zu beantragen ist unangenehm
- ☐ Eine Spielsperre ist nur für Spielsüchtige
- ☐ Eine Spielsperre kann man sowieso umgehen
- ☐ Ich wusste nicht, dass diese Möglichkeit besteht
- ☐ Keine Angabe
- ☐ Anderer Grund: ____

→ weiter mit **Frage D.13**

Für freiwillige Spielsperren:

D.9 Haben Sie sich zu früh, rechtzeitig oder zu spät sperren lassen?

☐ Zu früh ☐ Rechtzeitig ☐ Zu spät ☐ Keine Angabe

D.10 Aus welchem Hauptgrund haben Sie sich freiwillig sperren lassen? (Bitte geben Sie nur den Hauptgrund an)

- ☐ Präventiv
- ☐ Wunsch von Familie oder Freund/innen
- ☐ Zu viel Geld verloren
- ☐ Zu hohe Spieleinsätze riskiert, die nicht im Verhältnis zum Einkommen und Vermögen sind
- ☐ Schulden
- ☐ Finanzielle Probleme
- ☐ Zu viel Zeit im Casino verbracht
- ☐ Probleme in der Beziehung oder in der Familie
- ☐ Probleme bei der Arbeit
- ☐ Kontrollverlust über das Spielen
- ☐ Anregung einer Fachperson
- ☐ Eine nahestehende Person liess sich sperren
- ☐ Weil ich spielsüchtig bin
- ☐ Keine Angabe
- ☐ Anderer Grund: ____

D.11 Wie spontan war Ihr Entschluss, sich sperren zu lassen? *(1 bedeutet «überhaupt nicht spontan» und 10 bedeutet «völlig spontan»)*

überhaupt nicht spontan									völlig spontan
1	2	3	4	5	6	7	8	9	10
☐	☐	☐	☐	☐	☐	☐	☐	☐	☐

D.12 Wer hat die wichtigste Rolle bei Ihrem Entschluss gespielt, sich sperren zu lassen?

- ☐ Ich selber
- ☐ Angehörige, Freund/innen
- ☐ Lebenspartner/in
- ☐ Casinopersonal
- ☐ Berater/innen, Therapeut/innen
- ☐ Sonstige: ____
- ☐ Keine Angabe

Für freiwillige und angeordnete Spielsperren:

D.13 Sollte die Spielsperre aufgehoben werden: Schätzen Sie sich als gefährdet ein, wieder Spielprobleme zu entwickeln? *(0 bedeutet «überhaupt nicht gefährdet » und 10 bedeutet «sehr gefährdet»)*

überhaupt nicht gefährdet										sehr gefährdet
0	1	2	3	4	5	6	7	8	9	10
☐	☐	☐	☐	☐	☐	☐	☐	☐	☐	☐

D.14 Wurden Sie vom Casinopersonal wegen Ihres Spielverhaltens angesprochen?

☐ Ja ☐ Nein ☐ Keine Angabe

D.15 Sind Sie aktuell für das Glücksspiel in einem ausländischen Casino gesperrt?

☐ Ja ↓

☐ Nein → weiter mit **Frage D.17**

☐ Keine Angabe → weiter mit **Frage D.17**

D.16 In welchem Land bzw. welchen Ländern sind Sie gesperrt?

☐ Deutschland ☐ Italien ☐ Österreich

☐ Frankreich ☐ Liechtenstein ☐ Andere: ____________

D.17 Waren Sie früher schon einmal gesperrt?

☐ Ja → Wann wurde diese Spielsperre aufgehoben: ____________ Monat/Jahr (z. B. 12/2018)

☐ Nein

☐ Keine Angabe

E. Fragen zur Beratung

Die nächsten Fragen betreffen die Inanspruchnahme von Behandlungs- und Beratungsangeboten **aufgrund des Glückspiels**.

E.1 Wurden Sie von einem/einer Casinomitarbeiter/in auf Behandlungs- oder Beratungsangebote hingewiesen?

☐ Ja ☐ Nein ☐ Keine Angabe

E.2 Haben Sie wegen des Glücksspiels bereits einmal unterstützende Behandlungs- oder Beratungsangebote genutzt?

☐ Ja ↓

☐ Nein, noch nicht → weiter mit **Frage E.4**

☐ Nein, da ich keine Spielprobleme habe → weiter mit **Frage F.1**

☐ Keine Angabe → weiter mit **Frage F.1**

E.3 Welche der folgenden Beratungs- oder Behandlungsangebote haben Sie schon einmal wegen des Glücksspielens genutzt und wann war dies?

	In den letzten 6 Monaten	In der Zeit davor	Keine Nutzung
Selbsthilfegruppe	☐	☐	☐
Selbsthilfegruppe im Internet (Chat/Forum)	☐	☐	☐
Beratungsangebote im Internet	☐	☐	☐
Schuldenberatungsstelle	☐	☐	☐

	In den letzten 6 Monaten	In der Zeit davor	Keine Nutzung
Hausarzt/Hausärztin oder Allgemeine/r Arzt/Ärztin	☐	☐	☐
Psychotherapeut/in oder Psychiater/in	☐	☐	☐
Stationärer Aufenthalt in Spital / Klinik	☐	☐	☐
Suchtberatung	☐	☐	☐
Angehörige und oder Freund/innen	☐	☐	☐
Religiöse Würdenträger/innen (z.B. Pfarrer/in, Imam, Rabbi, etc.)	☐	☐	☐
Andere Hilfsangebote	☐	☐	☐

→ **weiter mit Frage F.1**

E.4 Sie haben keine Beratungs- oder Behandlungsangebote in Anspruch genommen. Bitte geben Sie an inwieweit die genannten Gründe auf Sie zutreffen:

	Trifft gar nicht zu	Trifft wenig zu	Trifft etwas zu	Trifft ziemlich zu	Trifft voll und ganz zu	keine Angabe
Ich wusste nicht, wohin ich gehen sollte um Hilfe zu bekommen.	☐	☐	☐	☐	☐	☐
Ich hatte mir Sorgen darüber gemacht, was andere über mich denken würden.	☐	☐	☐	☐	☐	☐
Ich glaubte nicht, dass mir eine Behandlung helfen würde.	☐	☐	☐	☐	☐	☐
Ich dachte, dass mich eine Behandlung zu viel Zeit und Energie kostet.	☐	☐	☐	☐	☐	☐
Ich dachte, dass ich damit alleine fertig werden würde.	☐	☐	☐	☐	☐	☐
Ich wollte mir nicht eingestehen, dass ich Hilfe brauche.	☐	☐	☐	☐	☐	☐
Ich hatte das Gefühl, dass das Spielen kein grosses Problem in meinem Leben darstellt.	☐	☐	☐	☐	☐	☐
Ich war zu stolz, um Hilfe in Anspruch zu nehmen.	☐	☐	☐	☐	☐	☐
Ich fühlte mich nicht in der Lage, meine Probleme mit anderen zu besprechen.	☐	☐	☐	☐	☐	☐
Ich wollte nicht, dass man mich als süchtig oder psychisch krank einstuft.	☐	☐	☐	☐	☐	☐
Mit professionellen Hilfeangeboten habe ich eher schlechte Erfahrungen gemacht.	☐	☐	☐	☐	☐	☐
Meine Familie und Freund/innen haben mich nicht ausreichend darin unterstützt, Hilfe zu suchen.	☐	☐	☐	☐	☐	☐
Auf Glücksspielprobleme spezialisierte Hilfsangebote gab es in meinem Wohnort nicht.	☐	☐	☐	☐	☐	☐
Ich hatte Angst davor, mich als Versager/in zu fühlen, wenn ich trotz Hilfe nicht vom Spielen loskomme.	☐	☐	☐	☐	☐	☐

F. Fragen zum Substanzkonsum

Mit den folgenden Fragen möchten wir einen Überblick über Ihren Alkoholkonsum und über den Konsum anderer Substanzen gewinnen, die Sie in den letzten 6 Monaten konsumiert haben.

F.1 Wie oft haben Sie in den letzten 6 Monaten Alkohol konsumiert?

- ☐ Nie → `weiter mit` **`Frage F.3`**
- ☐ 1 – 3 mal im Monat
- ☐ 3 – 4 mal pro Woche
- ☐ Täglich
- ☐ Weniger als einmal im Monat
- ☐ 1 – 2 mal pro Woche
- ☐ 5 – 6 mal pro Woche

F.2 Wie viel Alkohol konsumieren Sie an einem typischen Tag, an dem Sie Alkohol trinken?

____ Anzahl Glas / Gläser Wein (1 Glas = 1 dl)

____ Anzahl Biere (1 Bier = 3.3 dl)

____ Anzahl Glas / Gläser hochprozentiger Alkohol (1 Glas = 2 cl)

F.3 Wie oft haben Sie in den letzten 6 Monaten Zigaretten geraucht?

- ☐ Nie → `weiter mit` **`Frage F.5`**
- ☐ 1 – 3 mal im Monat
- ☐ 3 – 4 mal pro Woche
- ☐ Täglich
- ☐ Weniger als einmal im Monat
- ☐ 1 – 2 mal pro Woche
- ☐ 5 – 6 mal pro Woche

F.4 Während eines typischen Tages an dem Sie Zigaretten rauchen, wie viele rauchen Sie dann?

____ Anzahl Zigaretten pro Tag

F.5 Wie oft haben Sie in den letzten 6 Monaten Cannabis konsumiert?

- ☐ Nie
- ☐ 1 – 3 mal im Monat
- ☐ 3 – 4 mal pro Woche
- ☐ Täglich
- ☐ Weniger als einmal im Monat
- ☐ 1 – 2 mal pro Woche
- ☐ 5 – 6 mal pro Woche

F.6 Wie oft haben Sie in den letzten 6 Monaten andere illegale Substanzen konsumiert?

- ☐ Nie
- ☐ 1 – 3 mal im Monat
- ☐ 3 – 4 mal pro Woche
- ☐ Täglich
- ☐ Weniger als einmal im Monat
- ☐ 1 – 2 mal pro Woche
- ☐ 5 – 6 mal pro Woche

→ Welche? ____________________

Haben Sie noch irgendwelche Anregungen/Bemerkungen?

____

Ihr persönlicher Code

Um die Daten der drei Fragebögen personenbezogen zuordnen zu können und dabei dennoch ihre Anonymität zu wahren, verwenden wir statt Ihres Namens einen anonymen Code. Diesen kennt ausser Ihnen niemand. Sie müssen sich den Code nicht merken. Wir werden Sie bitten den gleichen Code auch an der 2. und 3. Befragung zu erstellen.

Dieser persönliche Code besteht aus einer Kombination von Buchstaben und Zahlen. Bitte geben Sie ...

- ... den letzten Buchstaben Ihres Geburtsmonates an. (Bsp.: Janua**r**)
- ... die ersten beiden Buchstaben des Vornamens Ihrer Mutter ein. (Bsp.: **An**na)
- ... die ersten beiden Buchstaben des Vornamens Ihres Vaters ein. (Bsp.: **Ma**rk)
- ... die dritte und vierte Stelle Ihres Geburtsjahres an. (Bsp.: 19**79**)

→ Aus dem Beispiel resultiert der Code «ranma79»

Bitte tragen Sie hier Ihren Code ein: ____________

Vielen Dank für Ihre Teilnahme! - Team Glücksspielstudie
