# Supplementary material for: Motivating factors and barriers to help-seeking for casino gamblers: results from a survey in Swiss casinos
Source: Front Psychiatry. 2023 May 25;14:1128291. doi: 10.3389/fpsyt.2023.1128291 (PMC10249729; doi:10.3389/fpsyt.2023.1128291)
Supplement: Supplementary file 3 [file Data_Sheet_3.PDF]

Lucerne University of
Applied Sciences and Arts

HOCHSCHULE
LUZERN

FH Zentralschweiz

FONDO NAZIONALE SVIZZERO
PER LA RICERCA SCIENTIFICA

Studio sulgioco

La ringraziamo per la sua preziosa collaborazione!

Jacqueline Mathys, Suzanne Lischer,
Angela Steffen, Jürg Schwarz

Lucerna I 12.08.2019

Modulo parte 1

Scuola Universitaria Professionale di Lucerna
Werftestrasse 1
Casella postale 2945
CH-6002 Lucerna

suzanne.lischer@hslu.ch

Istruzioni per riempire il modulo
Non dovrà rispondere a tutte le domande (a seconda della situazione). Questa possibilità è indicata con una freccia e con il numero della domanda a cui può passare. Ad esempio alla domanda A.3, se ha indicato come paese la Svizzera, può proseguire con la domanda A.5, senza rispondere alla domanda A.4.

Al fine di ottenere dei risultati significativi è importante che lei legga attentamente le domande, rispondendo, nel limite del possibile, a tutte. Nel caso in cui lei non desideri rispondere a una domanda potrà selezionare l'opzione « Non disponibile».

A. Domande sui dati personali

A.1 Indichi il suo sesso: ☐ Donna ☐ Uomo ☐ Diverso ☐ Non disponibile

A.2 Data di nascita ________ (Anno, p.es. 1966)

A.3 Paese di nascita ________ → Se indicato «Svizzera», continuare con la **domanda A.5**

A.4 Da quanto tempo vive in Svizzera? ________ (Anno, p.es. 1983)

A.5 Quale(i) cittadinanze(i) avete? *Se ne avete più di una vi preghiamo di indicare le due più importanti per voi.* ________

A.6 Qual è la cittadinanza di sua madre? ________ suo padre? ________

A.7 Qual è il suo stato di civile ufficiale?

- ☐ Celibe/nubile
- ☐ Coniugato / Unione domestica registrata
- ☐ Divorziato/a / Unione domestica registrata sciolta
- ☐ Ho una relazione
- ☐ Vedovo/a
- ☐ Non disponibile

A.8 Vive insieme a persone adulte?

- ☐ No
- ☐ Sì, in comunità domestica
- ☐ Altro
- ☐ Sì, con il/la coniuge o il/la convivente
- ☐ Sì, con i genitori, un genitore o congiunto
- ☐ Non disponibile

A.9 Ha figli soggetti all'obbligo di assistenza?

- ☐ Sì, numero: ____ e vive/vivono nella mia economia domestica.
- ☐ No
- ☐ Non disponibile
- ☐ Sì, numero: ____ ma non vive/vivono nella mia economia domestica.

A.10 Qual è la formazione di grado più elevato da lei conseguita?

- ☐ Nessun titolo di studio conseguito della scuola dell'obbligo
- ☐ Tirocinio o scuola professionale a tempo pieno, tirocinio con maturità professionale
- ☐ Università, scuola universitaria, scuola universitaria professionale
- ☐ Scuola dell'obbligo
- ☐ Scuola di diploma, maturità, scuola magistrale
- ☐ Altra formazione
- ☐ Formazione empirica, un anno di scuola di commercio, un anno di economia domestica
- ☐ Istituto tecnico, scuola specializzata superiore (STS/SSQEA), attestato professionale superiore
- ☐ Non disponibile

A.11 A quanto ammonta il suo reddito netto mensile personale (in CHF)?

- ☐ Meno di CHF 3'000
- ☐ CHF 7'001 – 9'000
- ☐ Più di CHF 13'000
- ☐ CHF 3'000 – 5'000
- ☐ CHF 9'001 – 11'000
- ☐ Non disponibile
- ☐ CHF 5'001 – 7'000
- ☐ CHF 11'001 – 13'000

A.12 Quale forma di attività lavorativa svolge attualmente principalmente?

- ☐ Indipendente
- ☐ Casalingo/a
- ☐ Studente/sa
- ☐ Dipendente
- ☐ Beneficiario/a di aiuti sociali
- ☐ Altro
- ☐ Pensionato/a di vecchiaia (AVS)
- ☐ Beneficiario/a AD
- ☐ Non disponibile
- ☐ Beneficiario/a AI
- ☐ In formazione, praticantato

B. Domande sul comportamento di gioco

Vorremmo ora porle alcune domande sul suo comportamento di gioco. Il termine generico di gioco definisce tutti i giochi e scommesse con denaro.

B.1 Ci indichi gentilmente quanto le seguenti affermazioni corrispondono al suo comportamento.	Non corrisponde affatto	Corrisponde poco	Corrisponde in parte	Corrisponde abbastanza	Corrisponde perfettamente	Non disponibile
Pratico / praticavo il gioco d'azzardo...						
... per divertimento	☐	☐	☐	☐	☐	☐
... per abitudine	☐	☐	☐	☐	☐	☐
... perché mi dà adrenalina	☐	☐	☐	☐	☐	☐
... perché lo fanno anche i miei amici	☐	☐	☐	☐	☐	☐
... per noia	☐	☐	☐	☐	☐	☐
... perché mi aiuta a dimenticare tutto il resto	☐	☐	☐	☐	☐	☐
... perché voglio vincere soldi	☐	☐	☐	☐	☐	☐
... per trovarmi più a mio agio con gli altri	☐	☐	☐	☐	☐	☐
... quando sono sono stressato/a o depresso/a o preoccupato/a	☐	☐	☐	☐	☐	☐
... per la sensazione di felicità, potere e successo	☐	☐	☐	☐	☐	☐
... per il prestigio e/o l'ambiente	☐	☐	☐	☐	☐	☐

B.2 Di seguito troverà un elenco **di giochi d'azzardo in Svizzera**, **di giochi d'azzardo all'estero** e **di giochi d'azzardo in Internet**.

Riferisca con quale frequenza e per quanto tempo ha giocato negli **ultimi 6 mesi**. **I/le giocatori/trici esclusi/e** sono pregati/e di fare riferimento ai **6 mesi precedenti l'esclusione**.

	Se lo ha praticato, quante volte è accaduto in un mese in media?							Ha giocato e in caso di risposta affermativa quante sono le ore di gioco?
**Giochi d'azzardo in Svizzera (offline)**	mai	Meno di una volta al mese	1–3 volte al mese	1–2 volte alla settimana	3–4 volte alla settimana	5–6 volte alla settimana	quotidianamente	Specifica in ore
Roulette, black jack, altri giochi da tavolo nei casinò (escluso il poker)	☐	☐	☐	☐	☐	☐	☐	___
Apparecchi automatici da gioco (slot machine) nei casinò	☐	☐	☐	☐	☐	☐	☐	___
Poker nei casinò	☐	☐	☐	☐	☐	☐	☐	___
Tornei di poker (nessun torneo di poker al di fuori dei casinò)	☐	☐	☐	☐	☐	☐	☐	___
Poker privato (amici e famiglia)	☐	☐	☐	☐	☐	☐	☐	___
Giochi d'azzardo nei sala del retro nei club, bar e locali del circolo	☐	☐	☐	☐	☐	☐	☐	___

	Se lo ha praticato, quante volte è accaduto in un mese in media?							Ha giocato e in caso di risposta affermativa quante sono le ore di gioco?
	mai	Meno di una volta al mese	1–3 volte al mese	1–2 volte alla settimana	3–4 volte alla settimana	5–6 volte alla settimana	quotidianamente	Specifica in ore
Poker nei sala del retro nei club, bar e locali del circolo	☐	☐	☐	☐	☐	☐	☐	
Scommesse sportive (calcio ecc.)	☐	☐	☐	☐	☐	☐	☐	
Scommesse sportive nei sala del retro nei club, bar e locali del circolo	☐	☐	☐	☐	☐	☐	☐	
Scommesse sui cavalli (Swisslos, LoRo)	☐	☐	☐	☐	☐	☐	☐	
Tactilo (lotterie elettroniche)	☐	☐	☐	☐	☐	☐	☐	
Lotto, lotteria all'edicola (Swisslos, LoRo)	☐	☐	☐	☐	☐	☐	☐	
Altri giochi d'azzardo	☐	☐	☐	☐	☐	☐	☐	
**Giochi d'azzardo all'estero (offline)**								
Giochi d'azzardo nei casinò all'estero	☐	☐	☐	☐	☐	☐	☐	
Sale giochi all'estero	☐	☐	☐	☐	☐	☐	☐	
Altri giochi d'azzardo	☐	☐	☐	☐	☐	☐	☐	
**Giochi d'azzardo in Internet**								
Giochi d'azzardo in Internet di operatori di casinò svizzeri	☐	☐	☐	☐	☐	☐	☐	
Giochi d'azzardo in Internet di operatori esteri	☐	☐	☐	☐	☐	☐	☐	
Poker in Internet di operatori di casinò svizzeri	☐	☐	☐	☐	☐	☐	☐	
Poker in Internet di altri operatori	☐	☐	☐	☐	☐	☐	☐	
Scommesse sportive in internet (Swisslos, LoRo)	☐	☐	☐	☐	☐	☐	☐	
Scommesse sportive in internet presso altri operatori	☐	☐	☐	☐	☐	☐	☐	
Lotto in Internet di operatori di casinò svizzeri (Swisslos, LoRo)	☐	☐	☐	☐	☐	☐	☐	
Speculazione in borsa o sul mercato delle opzioni	☐	☐	☐	☐	☐	☐	☐	
Altri giochi d'azzardo in internet	☐	☐	☐	☐	☐	☐	☐	

B.3 Quale dei giochi d'azzardo che figurano nell'elenco è stato di maggior interesse per lei negli ultimi 6 mesi?

B.4 La sapeva che dal 1° gennaio 2019 i casinò i casinò svizzeri propongono legalmente sui propri siti web anche i giochi d'azzardo online?

☐ Sì↓ ☐ No → `continuare con` **`la domanda B.8`**

☐ Non disponibile → `continuare con` **`la domanda B.8`**

B.5 Lei si avvale di tale offerta?

- ☐ Sì ↓
- ☐ No → continuare con **la domanda B.7**
- ☐ Non disponibile → continuare con **la domanda B.7**

B.6 Per quale motivo si avvale dell'offerta online dei casinò svizzeri?

- ☐ Perché è legale
- ☐ Massima fiducia nei casinò svizzeri
- ☐ Offerta allettante
- ☐ Non so
- ☐ Non disponibile
- ☐ Altro motivo: ____

B.7 Per quale motivo non si avvale della nuova offerta online dei casinò svizzeri?

- ☐ Non mi piacciono i giochi online
- ☐ Preferisco le offerte di altri operatori
- ☐ Non so
- ☐ Non disponibile
- ☐ Altro motivo: ____

B.8 Le è mai capitata una grossa vincita? (offline o online)

- ☐ No
- ☐ Sì, in totale circa: ____ CHF
- ☐ Non disponibile

B.9 Quale importo ha puntato mensilmente al gioco d'azzardo negli ultimi 6 mesi in media?

- ☐ Meno di CHF 10
- ☐ CHF 10 – 99
- ☐ CHF 100 – 299
- ☐ CHF 300 – 499
- ☐ CHF 500 – 999
- ☐ CHF 1000 – 2'499
- ☐ CHF 2'500 – 9'999
- ☐ CHF 10'000 o più
- ☐ Non so
- ☐ Non disponibile

B.10 Ha un tetto massimo mensile per le puntate ai giochi d'azzardo?

- ☐ Sì ↓
- ☐ No → continuare con **la domanda B.12**
- ☐ Non disponibile → continuare con **la domanda B.12**

B.11 Ha difficoltà a rispettare i limiti?

- ☐ Mai
- ☐ Raramente
- ☐ A volte
- ☐ Spesso
- ☐ Sempre
- ☐ Non disponibile

B.12 Negli ultimi 6 mesi è mai andato in prestito di denaro da qualcuno senza rimborsarlo, come conseguenza del suo gioco d'azzardo?

- ☐ Sì
- ☐ No

B.13 Negli ultimi 6 mesi, se lei si è fatto prestare del denaro per giocare d'azzardo o per pagare debiti di gioco d'azzardo, da chi o da dove li ha presi a prestito? (Sono possibili più risposte)

- ☐ Non ha preso in prestito denaro
- ☐ Dal denaro di famiglia
- ☐ Dal coniuge
- ☐ Da altri familiari o parenti acquisiti
- ☐ Da banche, da società di prestito o da compagnie di credito, ecc.
- ☐ Da carte di credito
- ☐ Da usurai
- ☐ Ha venduto titoli, obbligazioni o altri valori
- ☐ Ha venduto proprietà personali o della famiglia
- ☐ Ha preso denaro dal suo conto corrente (emettendo assegni a vuoto)
- ☐ Da amici
- ☐ Ha (o ha avuto) un credito da un allibratore

B.14 Se ha debiti dovuti al gioco d'azzardo, a quanto ammontano complessivamente?

- ☐ No
- ☐ Non lo so
- ☐ Non disponibile
- ☐ Sì, in totale circa: ____ CHF

B.15 Ha commesso atti illeciti come falsificazione, truffa, furto o defraudamento per finanziare il gioco d'azzardo o per pagare i debiti di gioco?

- ☐ Sì
- ☐ No
- ☐ Non disponibile

In alcuni soggetti possono manifestarsi dei problemi dovuti al gioco d'azzardo.
Vorremmo conoscere la sua situazione.

B.16 Negli ultimi 6 mesi, crede di aver avuto un problema con le scommesse o con il gioco d'azzardo?

- ☐ Sì ↓
- ☐ No → continuare con **la domanda B.18**

B.17 Da quanto, secondo lei, dura già questo problema?

- ☐ ______ anni
- ☐ Non so
- ☐ Non disponibile

B.18 Se ha giocato negli ultimi 6 mesi, quante volte le capita di ritornare a giocare nei giorni successivi per rivincere le somme perdute?

- ☐ Mai negli ultimi 6 mesi.
- ☐ La maggior parte delle volte in cui avevo perso
- ☐ Alcune volte (meno della metà delle volte in cui avevo perso)
- ☐ Tutte le volte in cui avevo perso

B.19 Negli ultimi 6 mesi ha mai affermato di aver vinto denaro al gioco d'azzardo quando non era vero, avendo perso in realtà?

- ☐ Mai
- ☐ Sì, meno della metà delle volte in cui avevo perso
- ☐ Sì, per la maggior parte delle volte

Le seguenti domande si riferiscono agli ultimi 6 mesi.

	Sì	No
B.20 Ha giocato più di quanto aveva preventivato?	☐	☐
B.21 Ha ricevuto critiche da altre persone riguardo al suo comportamento di gioco o qualcuno le ha detto che soffre di dipendenza da gioco d'azzardo, indipendentemente dal fatto che sia d'accordo o meno?	☐	☐
B.22 Si sentiva in colpa per il modo con cui gioca o per quanto accade quando gioca?	☐	☐
B.23 Avete pensato che vorrebbe smettere di scommettere denaro o di giocare d'azzardo, pensando però di non riuscirci?	☐	☐
B.24 Ha nascosto scontrini di scommesse, biglietti di lotteria, denaro vinto al gioco, riconoscimenti di debiti o altre prove di scommessa o di gioco d'azzardo al coniuge, ai figli o ad altre persone significative nella sua vita?	☐	☐
B.25 Hai saltato il assente dal lavoro (o da scuola) per scommettere denaro o per giocare d'azzardo?	☐	☐
B.26 Hai discusso con le persone con cui vive su come utilizza il denaro?	☐	☐
Se ha risposto sì: I litigi sul denaro sono rnai stati concentrati sul suo gioco d'azzardo?	☐	☐

B.27 Nel suo ambiente sociale ci sono altre persone che hanno o hanno avuto **problemi di gioco**?

- ☐ Sì ↓
- ☐ No → continuare con **la domanda C.1**
- ☐ Non so → continuare con **la domanda C.1**
- ☐ Non disponibile → continuare con **la domanda C.1**

B.28 Indichi chi delle seguenti persone nella loro vita ha (o ha avuto) un problema con il gioco d'azzardo.

- ☐ Coniuge/partner
- ☐ Padre
- ☐ Figli
- ☐ Amici
- ☐ Madre
- ☐ Fratelli e sorelle
- ☐ Altri congiunti che le sono vicini

C. La domande sulla qualità della vita

C.1 Le seguenti affermazioni riguardano il vostro benessere nelle ultime 2 settimane. Per ogni affermazione, scegliete la risposta che ritenete più adatta a descrivere come vi siete sentiti durante le ultime due settimane.

Nelle ultime 2 settimane ...	Tutto il tempo	La maggior parte del tempo	Un po' più della metà del tempo	Un po' meno della metà del tempo	Di tanto in tanto	In nessun momento
... Mi sono sentito/a allegro/a e di buon umore	☐	☐	☐	☐	☐	☐
... Mi sono sentito/a calmo/a e rilassato/a	☐	☐	☐	☐	☐	☐
... Mi sono sentito/a attivo/a ed energico/a	☐	☐	☐	☐	☐	☐
... Mi sono svegliato/a sentendomi fresco/a e riposato/a	☐	☐	☐	☐	☐	☐
... La mia vita di tutti i giorni è stata piena di cose che mi interessano	☐	☐	☐	☐	☐	☐

C.2 Quanto corrispondono le seguenti affermazioni al suo modo di agire e al suo comportamento?

	Non corrisponde per niente	Piuttosto non corrisponde	Corrisponde in parte sì e in parte no	Corrisponde abbastanza	Corrisponde completamente	Non disponibile
In situazioni difficili posso fidarmi delle mie capacità	☐	☐	☐	☐	☐	☐
Posso superare la maggior parte dei problemi con le mie proprie forze	☐	☐	☐	☐	☐	☐
Di regola riesco a risolvere anche compiti impegnativi e complicati	☐	☐	☐	☐	☐	☐

C.3 Quanto spesso nelle ultime due settimane ha avvertito i seguenti disturbi?

	Mai	Alcuni giorni	Per più della metà del tempo	Quasi ogni giorno	Non disponibile
Scarso interesse o piacere nel fare le cose	☐	☐	☐	☐	☐
Sentirsi giù di morale, depresso o disperato	☐	☐	☐	☐	☐
Sentirsi nervoso/a, ansioso/a o teso/a	☐	☐	☐	☐	☐
Non riuscire a smettere di preoccuparsi o a tenere sotto controllo le preoccupazioni	☐	☐	☐	☐	☐

C.4 In linea di massima, in che misura Lei è soddisfatto della Sua vita? *(0 significa "perniente soddisfatto" e 10 "pienamente soddisfatto".)*

perniente soddisfatto — pienamente soddisfatto

0	1	2	3	4	5	6	7	8	9	10
☐	☐	☐	☐	☐	☐	☐	☐	☐	☐	☐

C.5 Indichi quanto si ritiene soddisfatto/a nelle seguenti sfere della vita:
(0 significa "per niente soddisfatto" e 10 "pienamente soddisfatto")

Qual è globalmente il Suo grado ...	0 (per niente soddisfatto)	1	2	3	4	5	6	7	8	9	10 (pienamente soddisfatto)
... situazione finanziaria?	☐	☐	☐	☐	☐	☐	☐	☐	☐	☐	☐
... relazioni personali familiari e sociali?	☐	☐	☐	☐	☐	☐	☐	☐	☐	☐	☐
... con il tuo tempo libero?	☐	☐	☐	☐	☐	☐	☐	☐	☐	☐	☐
... situazione abitativa?	☐	☐	☐	☐	☐	☐	☐	☐	☐	☐	☐
... E' soddisfatto(a) della Sua salute?	☐	☐	☐	☐	☐	☐	☐	☐	☐	☐	☐
... situazione lavorativa?	☐	☐	☐	☐	☐	☐	☐	☐	☐	☐	☐

D. Domande sull’esclusione dal gioco

D.1 In questo momento lei è escluso/a dal gioco d’azzardo in un casinò svizzero?

Domanda obbligatoria! Questa domanda è di grande importanza. Si prega di rispondere.

☐ Sì ↓ ☐ No → continuare con **la domanda D.14**

D.2 Quando è stata effettuata l’esclusione? **Domanda obbligatoria!**

__________ Data (a. e. 20.2.2019)

D.3 Quale offerta è stata la ragione del divieto di gioco? **Domanda obbligatoria!**

☐ Gioco d'azzardo da casinò ☐ Gioco d'azzardo in Internet ☐ Swisslos, LoRo

D.4 Di quale tipo di esclusione si tratta? **Domanda obbligatoria!**

☐ Esclusione dal gioco imposta ↓

☐ Esclusione dal gioco volontaria → continuare con **la domanda D.9**

Per Esclusione dal gioco imposta:

D.5 A Suo avviso Lei è stato/a escluso/a dal gioco troppo presto, al momento giusto o troppo tardi?

☐ Troppo presto ☐ Al momento giusto ☐ Troppo tardi ☐ Non disponibile

D.6 Per quale motivo è stata ordinata l’esclusione?

☐ Non è stata fornita alcuna giustificazione finanziaria ☐ Segnalazione di terzi ☐ Altro motivo, in particolare

☐ Segnalazione autorità sociali, servizio specializzato ☐ Esclusione per attività fraudolente ☐ Altro motivo: __________

D.7 A suo avviso, l’esclusione dal gioco era giustificata?

☐ Sì ☐ No ☐ Non disponibile

D.8 Perché non ha richiesto un'esclusione dal gioco volontaria? (Si prega di specificare una sola risposta)

- ☐ Non ci sono motivi per un'esclusione dal gioco.
- ☐ Volevo continuare a praticare il gioco d'azzardo.
- ☐ Volevo innanzitutto recuperare il denaro perso.
- ☐ È spiacevole richiede un'esclusione dal gioco volontaria.
- ☐ L'esclusione dal gioco è solo per coloro che hanno una dipendenza dal gioco.
- ☐ Un'esclusione dal gioco è comunque aggirabile.
- ☐ Non sapevo che esistesse questa possibilità.
- ☐ Non disponibile
- ☐ Altro motivo: ____

→ continuare con **la domanda D.13**

Per esclusione dal gioco volontaria:

D.9 Si è fatto/a escludere dal gioco troppo presto, al momento giusto o troppo tardi?

☐ Troppo presto ☐ Al momento giusto ☐ Troppo tardi ☐ Non disponibile

D.10 Per quale motivo ha richiesto un'esclusione volontaria? (Si prega di specificare una sola risposta)

- ☐ Prevenzione
- ☐ Desiderio di familiari o amici
- ☐ Somma di denaro eccessiva persa al gioco
- ☐ Puntate al gioco eccessive rispetto a reddito e patrimonio
- ☐ Debiti
- ☐ Problemi finanziari
- ☐ Troppo tempo trascorso al casinò
- ☐ Problemi familiari o relazionali
- ☐ Problemi al lavoro
- ☐ Perdita di controllo rispetto al gioco
- ☐ Su stimolo di un esperto
- ☐ Una persona vicina si è fatta escludere
- ☐ Ho una dipendenza dal gioco
- ☐ Non disponibile
- ☐ Altro motivo: ____

D.11 Quanto è stata spontanea la sua decisione di farsi escludere? *(0 significa "per affatto spontanea" e 10 "totalmente spontanea")*

per affatto spontanea									totalmente spontanea
1	2	3	4	5	6	7	8	9	10
☐	☐	☐	☐	☐	☐	☐	☐	☐	☐

D.12 Chi ha avuto il ruolo principale nella decisione di farsi escludere?

- ☐ Io stesso/a
- ☐ Convivente
- ☐ Conoscenti / amici
- ☐ Personale del casinò
- ☐ Consulente / terapeuta
- ☐ Altro: ____
- ☐ Non disponibile

Per esclusione dal gioco volontaria e imposta:

D.13 Se l'esclusione dal gioco fosse revocata, pensa di essere a rischio di nuovi problemi con il gioco? *(0 significa "per affatto a rischio" e 10 "Molto a rischio")*

per affatto a rischio										Molto a rischio
0	1	2	3	4	5	6	7	8	9	10
☐	☐	☐	☐	☐	☐	☐	☐	☐	☐	☐

D.14 Il personale del casinò si è occupato di lei per via del suo comportamento di gioco?

☐ Sì ☐ No ☐ Non disponibile

D.15 In questo momento lei è escluso/a dal gioco d'azzardo in un casinò estero?

☐ Sì ↓
☐ No → continuare con **la domanda D.17**
☐ Non disponibile → continuare con **la domanda D.17**

D.16 In quale/i paese/i è escluso/a?

☐ Germania ☐ Italia ☐ Austria
☐ Francia ☐ Liechtenstein ☐ Altri: ______

D.17 Era già stato/a escluso/a prima di allora?

☐ Sì → Quando è stata revocata? ______ mese/anno (ad es 12/2018)
☐ No
☐ Non disponibile

E. Domande sulla consulenza

Le prossime domande riguardano il ricorso alle offerte di trattamento o consulenza **per via del gioco d'azzardo**.

E.1 Alcun collaboratore di casinò le ha consigliato di avvalersi di offerte di trattamento o di consulenza?

☐ Sì ☐ No ☐ Non disponibile

E.2 A causa del gioco d'azzardo si è già avvalso/a delle offerte di sostegno per ricevere trattamenti e consulenza?

☐ Sì ↓
☐ No, non ce l'ho ancorat → continuare con **la domanda E.4**
☐ No, perché non ho problemi con il gioco → continuare con **la domanda F.1**
☐ Non disponibile → continuare con **la domanda F.1**

E.3 Indichi se a causa del gioco d'azzardo si è avvalso/a una o più volte delle seguenti offerte di trattamento o consulenza e quando?

	Negli ultimi 6 mesi	Nel tempo prima di quello	Nessun uso
Gruppo di autoaiuto	☐	☐	☐
Gruppo di autoaiuto in Internet (chat / forum)	☐	☐	☐
Offerte di consulenza in Internet	☐	☐	☐
Ufficio di consulenza in caso di debiti	☐	☐	☐
Medico di famiglia / medico generico	☐	☐	☐

	Negli ultimi 6 mesi	Nel tempo prima di quello	Nessun uso
Psicologo/a / psicoterapeuta o psichiatra	☐	☐	☐
Degenza stazionaria in ospedale / clinica	☐	☐	☐
Consulenza per la dipendenze	☐	☐	☐
Parenti e amici	☐	☐	☐
Autorità religiose (ad es. parroco, imam, rabbino ecc.)	☐	☐	☐
Altre offerte di assistenza	☐	☐	☐

→ **continuare con la domanda F.1**

E.4 Perché non è ricorso/a ad alcuna offerta di trattamento o consulenza? Indichi cortesemente quale dei motivi elencati corrisponde al suo caso personale.

	Non corri-sponde	Corri-sponde abba-stanza poco	Corri-sponde in parte	Corri-sponde	Corri-sponde perfet-tamen-te	Non disponi-bile
Non sapevo a chi rivolgermi per chiedere aiuto.	☐	☐	☐	☐	☐	☐
Mi sono preoccupato di quello che avrebbero pensato gli altri di me.	☐	☐	☐	☐	☐	☐
Ho pensato che un trattamento non mi sarebbe stato di aiuto.	☐	☐	☐	☐	☐	☐
Ho ritenuto che un trattamento mi sarebbe costato troppo tempo ed energia.	☐	☐	☐	☐	☐	☐
Mi sono detto che potevo cavarmela da solo/a.	☐	☐	☐	☐	☐	☐
Non volevo ammettere con me stesso di aver bisogno di aiuto.	☐	☐	☐	☐	☐	☐
Ho avuto la sensazione che il gioco non costituisse un grande problema per la mia vita.	☐	☐	☐	☐	☐	☐
Ero troppo orgoglioso/a per accettare di ricevere aiuto.	☐	☐	☐	☐	☐	☐
Non mi sentivo in grado di discutere i miei problemi con gli altri.	☐	☐	☐	☐	☐	☐
Non volevo essere classificato/a come dipendente o affetto/a da una malattia psichica.	☐	☐	☐	☐	☐	☐
Ho avuto esperienze piuttosto negative con le offerte di assistenza.	☐	☐	☐	☐	☐	☐
La mia famiglia e gli amici non mi hanno incoraggiato abbastanza a cercare aiuto.	☐	☐	☐	☐	☐	☐
Nel mio luogo di domicilio non esistevano offerte di assi-stenza specializzate in problemi con il gioco d'azzardo.	☐	☐	☐	☐	☐	☐
Avevo paura di sentirmi fallito/a se non fossi riuscito/a a liberarmi del gioco nonostante l'aiuto.	☐	☐	☐	☐	☐	☐

F. Domande sull'assunzione di sostanze

Spesso, in relazione al gioco d'azzardo, emergono problemi legati a un maggiore consumo di sostanze come alcol o di altro genere. Con le seguenti domande desideriamo farci un'idea del consumo di alcol e altre sostanze da parte sua negli ultimi 6 mesi.

F.1 Con quale frequenza ha consumato alcolici negli ultimi sei mesi?

- ☐ Mai → `continuare con` **`la domanda F.3`**
- ☐ meno di una volta al mese
- ☐ 1 – 3 volte al mese
- ☐ 1 – 2 volte alla settimana
- ☐ 3 – 4 volte alla settimana
- ☐ 5 – 6 volte alla settimana
- ☐ quotidianamente

F.2 Quante bevande alcoliche consuma in un giorno normalmente quando beve?

- ____ numero di bicchiere di vino (1 bicchiere = 1 dl di vino)
- ____ numero di birre (una birra = 3,3 dl)
- ____ un bicchiere di superalcolici (1 bicchiere = 2 cl di superalcolici)

F.3 Con quale frequenza ha fumato sigarette negli ultimi 6 mesi?

- ☐ Mai → `continuare con` **`la domanda F.5`**
- ☐ meno di una volta al mese
- ☐ 1 – 3 volte alla settimana
- ☐ 1 – 2 volte alla settimana
- ☐ 3 – 4 volte alla settimana
- ☐ 5 – 6 volte alla settimana
- ☐ quotidianamente

F.4 In una giornata in cui fuma sigarette, quante ne fuma normalmente?

Quant. ____

F.5 Con quale frequenza ha consuma cannabis negli ultimi 6 mesi?

- ☐ Mai
- ☐ meno di una volta al mese
- ☐ 1 – 3 volte alla settimana
- ☐ 1 – 2 volte alla settimana
- ☐ 3 – 4 volte alla settimana
- ☐ 5 – 6 volte alla settimana
- ☐ quotidianamente

F.6 Con quale frequenza ha consuma altre sostanze illegali ultimi 6 mesi?

- ☐ Mai
- ☐ meno di una volta al mese
- ☐ 1 – 3 volte alla settimana
- ☐ 1 – 2 volte alla settimana
- ☐ 3 – 4 volte alla settimana
- ☐ 5 – 6 volte alla settimana
- ☐ quotidianamente

→ Quali sostanze? ____

Se desidera comunicarci qualcosa a proposito di questo studio o in generale sul gioco d'azzardo, può farlo utilizzando questo spazio.

____

Il suo codice personale
Per attribuire i dati dei tre moduli alla stessa persona e garantire comunque l'anonimato, invece di utilizzare il suo nome utilizziamo un codice anonimo. Nessuno, eccetto lei, conosce questo codice. Non occorre che si ricordi il codice. Le chiederemo di generare lo stesso codice anche al 2º e 3º modulo.

Questo codice personale è costituito da una combinazione di lettere e numeri. Si prega di inserire ...

- ... l'ultima lettera del suo mese di nascita. (Es.: gennai**o**)
- ... le prime due lettere del nome di sua madre. (Es.: **An**na)
- ... le prime due lettere del nome di suo padre. (Es.: **Ma**rco)
- ... la terza e quarta cifra del suo anno di nascita. (Es.: 19**79**)

→ Dall'esempio risulta il codice «oanma79»

Inserisca il **suo codice**: ____

Grazie per le sue preziose informazioni! – Team Studio sul gioco
