# Supplementary material for: Motivating factors and barriers to help-seeking for casino gamblers: results from a survey in Swiss casinos
Source: Front Psychiatry. 2023 May 25;14:1128291. doi: 10.3389/fpsyt.2023.1128291 (PMC10249729; doi:10.3389/fpsyt.2023.1128291)
Supplement: Supplementary file 5 [file Data_Sheet_5.PDF]

Lucerne University of
Applied Sciences and Arts
HOCHSCHULE
LUZERN
FH Zentralschweiz

FONDS NATIONAL SUISSE
DE LA RECHERCHE SCIENTIFIQUE

Étude sur le jeu

Merci pour votre précieux soutien!

Jacqueline Mathys, Suzanne Lischer,
Angela Steffen, Jürg Schwarz

Lucerne I 12.08.2019

Questionnaire partie 2

Haute école spécialisée de Lucerne
Werftestrasse 1
Case postale 2945
CH-6002 Lucerne

Suzanne.Lischer@hslu.ch

Instructions de remplissage

Selon votre situation, il se peut que vous n'ayez pas à répondre à toutes les questions. Nous l'avons marqué avec une flèche et le numéro de la question à laquelle vous pouvez accéder. Par exemple, pour la question A.1, si vous avez indiqué la Suisse ici, vous pouvez continuer avec la question A.3 et sauter la question A.2.

Pour obtenir des résultats significatifs, il est important que vous lisiez attentivement les questions et, si possible, que vous répondiez à toutes les questions. Si vous ne souhaitez pas répondre à une question, vous pouvez sélectionner l'option « aucune réponse ».

A. Questions sur votre personne

A.1 Votre état civil ou votre statut relationnel a-t-il changé au cours des 6 derniers mois ?

- ☐ Oui ↓
- ☐ Non → continuer avec **la question A.3**
- ☐ Aucune réponse → continuer avec **la question A.3**

A.2 Quel est votre nouvel état civil ?

- ☐ Célibataire
- ☐ Marié(e) / En partenariat enregistré
- ☐ Divorcé(e) / En partenariat enregistré dissous
- ☐ En couple
- ☐ Veuf ou veuve
- ☐ Aucune réponse

A.3 Y a-t-il eu des changements au niveau de votre activité professionnelle au cours des 6 derniers mois ?

- ☐ Oui ↓
- ☐ Non → continuer avec **la question A.5**
- ☐ Aucune réponse → continuer avec **la question A.5**

A.4 Quelle activité principale exercez-vous actuellement ?

- ☐ Statut d'indépendant
- ☐ Salarié(e)
- ☐ Retraité(e) (AVS)
- ☐ Bénéficiaire de l'AI
- ☐ Femme / homme au foyer
- ☐ Bénéficiaire de l'aide sociale
- ☐ Bénéficiaire de l'assurance chômage
- ☐ En formation, en stage
- ☐ Étudiant(e)
- ☐ Autre
- ☐ Aucune réponse

A.5 Quel est votre revenu mensuel net personnel ?

- ☐ Moins de CHF 3'000
- ☐ CHF 3'000 – 5'000
- ☐ CHF 5'001 – 7'000
- ☐ CHF 7'001 – 9'000
- ☐ CHF 9'001 – 11'000
- ☐ CHF 11'001 – 13'000
- ☐ Plus de CHF 13'000
- ☐ Aucune réponse

A.6 Vivez-vous en Suisse ?

- ☐ Oui
- ☐ Non
- ☐ Aucune réponse

A.7 Travaillez-vous en Suisse ?

- ☐ Oui
- ☐ Oui, mais seulement de façon saisonnière
- ☐ Non
- ☐ Aucune réponse

B. Question sur votre comportement de jeu

Les questions qui suivent portent sur votre comportement de jeu au cours des 6 derniers mois. Le terme générique de jeu désigne tous les jeux et paris avec de l'argent.

B.1 Avez-vous participé à des jeux de hasard au cours des 6 derniers mois ? (Par ex. à la roulette, au poker ou au lotto)

☐ Qui ↓
☐ No → continuer avec **la question B.9**

B.2 Vous trouverez ci-dessous une liste des **jeux de hasard en Suisse**, des **jeux de hasard à l'étranger** et des **jeux de hasard sur internet**.

Veuillez indiquer combien de fois et combien de temps vous avez participé **au cours des 6 derniers mois**. Si vous faites partie des joueurs/joueuses étant actuellement ou ayant été exclus des jeux au cours des 6 derniers mois, veuillez répondre **en pensant à cette période d'exclusion**.

	Combien de fois par mois en moyenne y avez-vous joué ?							Combien d'heures avez-vous passé à chaque fois que vous avez joué ?
	Ja-mais	Moins d'une fois par mois	1–3 fois par mois	1–2 fois par se-maine	3–4 fois par se-maine	5–6 fois par se-maine	Tous les jours	Spécifi-cations en h
**Jeux de hasard en Suisse (offline)**								
La roulette, le Black Jack, d'autres jeux de table au casino (excepté le poker)	☐	☐	☐	☐	☐	☐	☐	___
Les machines à sous (« slots ») au casino	☐	☐	☐	☐	☐	☐	☐	___
Le poker au casino	☐	☐	☐	☐	☐	☐	☐	___
Les tournois de poker (petits tournois de poker à l'extérieur)	☐	☐	☐	☐	☐	☐	☐	___
Le poker en milieu privé (ami(e)s et membres de la famille)	☐	☐	☐	☐	☐	☐	☐	___
Jeux de hasard dans « l'arrière-salle » dans les clubs, les bars et les locaux associatifs	☐	☐	☐	☐	☐	☐	☐	___
Poker dans « l'arrière-salle » dans les clubs, les bars et les locaux associatifs	☐	☐	☐	☐	☐	☐	☐	___
Les paris sportifs (football, etc.)	☐	☐	☐	☐	☐	☐	☐	___
Paris sportifs dans « l'arrière-salle » dans les clubs, les bars et les locaux associatifs	☐	☐	☐	☐	☐	☐	☐	___
Les courses de chevaux (Swisslos, LoRo)	☐	☐	☐	☐	☐	☐	☐	___
Tactilo (loteries électroniques)	☐	☐	☐	☐	☐	☐	☐	___
Le loto, les billets à gratter au kiosque (Swisslos, LoRo)	☐	☐	☐	☐	☐	☐	☐	___
Autres jeux de hasard	☐	☐	☐	☐	☐	☐	☐	___
**Jeux de hasard à l'étranger (offline)**								
Les jeux de hasard au casino à l'étranger	☐	☐	☐	☐	☐	☐	☐	___
Les arcades à l'étranger	☐	☐	☐	☐	☐	☐	☐	___
Autres jeux de hasard	☐	☐	☐	☐	☐	☐	☐	___

Jeu de hazard en ligne	Jamais	Moins d'une fois par mois	1–3 fois par mois	1–2 fois par semaine	3–4 fois par semaine	5–6 fois par semaine	Tous les jours	Spécifications en h
	Combien de fois par mois en moyenne y avez-vous joué ?							Combien d'heures avez-vous passé à chaque fois que vous avez joué ?
Jeux de hasard sur les sites internet des casinos suisses	☐	☐	☐	☐	☐	☐	☐	____
Jeux de hasard sur les sites internet d'autres prestataires	☐	☐	☐	☐	☐	☐	☐	____
Poker sur les sites internet des casinos suisses	☐	☐	☐	☐	☐	☐	☐	____
Poker sur les sites internet des autres prestataires	☐	☐	☐	☐	☐	☐	☐	____
Paris sportifs sur internet (Swisslos, LoRo)	☐	☐	☐	☐	☐	☐	☐	____
Paris sportifs sur les sites internet d'autres prestataires	☐	☐	☐	☐	☐	☐	☐	____
Loto sur internet (Swisslos, LoRo)	☐	☐	☐	☐	☐	☐	☐	____
«Spéculations» à la bourse ou sur le marché des options	☐	☐	☐	☐	☐	☐	☐	____
Autres jeux de hasard sur internet	☐	☐	☐	☐	☐	☐	☐	____

B.3 Lequel des jeux de hasard que vous avez cités a été le plus important pour vous au cours des 6 derniers mois ? ____________________

B.4 Veuillez indiquer dans quelle mesure vous êtes d'accord avec les énoncés suivants.	Pas du tout d'accord	Pas tout à fait d'accord	Plutôt d'accord	Assez d'accord	Tout à fait d'accord	Aucune réponse
Je joue / jouais aux jeux de hasard …						
… parce que cela me donne du plaisir	☐	☐	☐	☐	☐	☐
… par habitude	☐	☐	☐	☐	☐	☐
… parce que cela me donne des sensations fortes	☐	☐	☐	☐	☐	☐
… parce que mes ami(e) s jouent aussi	☐	☐	☐	☐	☐	☐
… lorsque je m'ennuie	☐	☐	☐	☐	☐	☐
… parce que cela me permet de ne pas penser à ce qui se passe dans mon entourage	☐	☐	☐	☐	☐	☐
… parce que j'aimerais gagner de l'argent	☐	☐	☐	☐	☐	☐
… parce que c'est si facile d'interagir avec les gens dans un lieu de jeu	☐	☐	☐	☐	☐	☐
… quand je suis/étais stressé(e), déprimé(e) ou que j'ai/avais des soucis	☐	☐	☐	☐	☐	☐
… en raison du sentiment de joie et de réussite que cela me procure	☐	☐	☐	☐	☐	☐
… pour le prestige et/ou à cause de l'ambiance	☐	☐	☐	☐	☐	☐

B.5 Vous est-il arrivé de gagner une grosse somme d'argent au course des 6 derniers mois ? (offline ou online)

☐ Non ☐ Oui, à savoir: ________ CHF ☐ Aucune réponse

B.6 Au cours des 6 derniers mois, quelle somme avez-vous dépensée en moyenne par mois pour des jeux de hasard ?

☐ Moins de CHF 10 ☐ CHF 300 – 499 ☐ CHF 2500 – 9'999 ☐ Aucune réponse

☐ CHF 10 – 99 ☐ CHF 500 – 999 ☐ CHF 10'000 ou plus

☐ CHF 100 – 299 ☐ CHF 1000 – 2'499 ☐ Je ne sais pas

B.7 Vous êtes-vous fixé un montant limite à miser dans les jeux de hasard par mois ?

☐ Oui↓ ☐ Non → continuer avec **la question B.9**

☐ Aucune réponse → continuer avec **la question B.9**

B.8 Avez-vous des difficultés à respecter cette limite ?

☐ Jamais ☐ Rarement ☐ Parfois ☐ Souvent ☐ Toujours ☐ Aucune réponse

B.9 Au cours des 6 derniers mois, avez-vous emprunté de l'argent à quelqu'un sans pouvoir le rembourser parce que vous aviez joué ?

☐ Oui
☐ Non

B.10 Si vous avez déjà emprunté de l'argent pour jouer ou pour rembourser des dettes de jeu, à qui l'avez-vous emprunté ? (plusieurs réponses possibles)

☐ Je n'ai pas emprunté d'argent ☐ D'une banque, d'une société de crédit ou d'une institution de prêt ☐ De la fortune/vente de proprié-té(s) personnelle(s) ou fami-liale(s)

☐ De votre budget familial ☐ De cartes de crédit ☐ En faisant de faux chèques (p. ex. émission de chèques sans provision)

☐ De votre conjoint(e), de votre petit(e)ami(e) ☐ De prêts usuriers (Shylocks) ☐ D'ami(e)s

☐ De membres de votre famille ou de votre belle-famille ☐ De vente d'actions, de bons d'épargne ou d'autres valeurs ☐ J'ai/j'avais un crédit de la part d'un(e) bookmaker

B.11 Avez-vous des dettes à cause des jeu de hasard ?

☐ Non ☐ Je ne sais pas ☐ Aucune réponse

☐ Oui, total env.: ________ CHF

B.12 Avez-vous commis des infractions (telles que la contrefaçon, la fraude, le vol ou le détournement de fonds) dans le but de financer votre activité de jeu de hazard ou de rembourser vos dettes de jeu?

☐ Oui
☐ Non
☐ Aucune réponse

Certaines personnes rencontrent des problèmes en raison de leur pratique des jeux de hasard. Nous aimerions savoir ce qu'il en est pour vous.

B.13 Pensez-vous avoir eu un problème de jeu au cours des 6 derniers mois ?

☐ Oui ↓ ☐ Non → continuer avec **la question B.15**

B.14 Depuis quand estimez-vous que ce problème existe ?

☐ ________ an(s) ☐ Je ne sais pas ☐ Aucune réponse

B.15 Lorsque vous avez joué au cours des 6 derniers mois, à quelle fréquence êtes-vous retourné(e) jouer le lendemain pour essayer de gagner à nouveau l'argent perdu la veille ?

☐ Jamais durant les 6 derniers mois ☐ La plupart des fois où j'ai perdu

☐ Quelquefois (moins de la moitié du nombre de fois où j'ai perdu) ☐ À Chaque fois que j'ai perdu

B.16 Au cours des 6 derniers mois, avez-vous déjà annoncé à tort que vous aviez gagné de l'argent, alors que ce n'était pas le cas en réalité?

☐ Jamais durant les 6 derniers mois ☐ Oui, moins de la moitié du nombre de fois où j'ai perdu ☐ Oui, la plupart des fois où j'ai perdu

Les questions suivantes se réfèrent aux 6 derniers mois.

	Oui	Non
B.17 Avez-vous déjà joué plus que vous ne l'aviez prévu ?	☐	☐
B.18 Les gens ont-ils critiqué le fait que vous jouiez ou vous ont-ils dit que vous aviez un problème avec le jeu, que cela soit vrai ou pas ?	☐	☐
B.19 Vous êtes-vous déjà senti coupable au sujet de la manière dont vous jouez ou des conséquences de votre jeu ?	☐	☐
B.20 Avez-vous ressenti l'envie d'arrêter de jouer et avez-vous pensé que vous n'y arriveriez pas ?	☐	☐
B.21 Avez-vous caché des bordereaux de paris, des billets de loterie, de l'argent pour parier, des reconnaissances de dette ou d'autres signes du jeu à votre épouse ou à votre mari, à vos enfants ou à une autre personne importante dans votre vie ?	☐	☐
B.22 Avez-vous manqué des heures de travail ou de cours pour jouer de l'argent ?	☐	☐
B.23 Vous êtes-vous disputé(e) avec une ou des personnes avec qui vous vivez au sujet de la façon dont vous gériez l'argent ?	☐	☐
Si oui, est-ce que ces disputes portaient sur vos habitudes de jeu ?	☐	☐

B.24 Avez-vous été confronté(e) à des conséquences négatives liées aux jeux de hasard au cours des 6 derniers mois ?

☐ Oui, à savoir: ____________________

☐ Non ☐ Aucune réponse

C. Questions sur la qualité de vie

C.1 Les déclarations suivantes concernent votre bien-être au cours des deux dernières semaines. Pour chaque énoncé, veuillez choisir la réponse qui, à votre avis, décrit le mieux ce que vous avez ressenti au cours des deux dernières semaines.

Au cours des 2 dernières semaines …	Tout le temps	La plupart du temps	Plus de la moitié du temps	Moins de la moitié du temps	De temps en temps	Jamais
… je me suis senti(e) bien et de bonne humeur	☐	☐	☐	☐	☐	☐
… je me suis senti(e) calme et tranquille.	☐	☐	☐	☐	☐	☐
… je me suis senti(e) plein(e) d'énergie et vigoureux(se)	☐	☐	☐	☐	☐	☐
… je me suis réveillé(e) en me sentant frais/fraîche et dispos(e)	☐	☐	☐	☐	☐	☐
… ma vie quotidienne a été remplie de choses intéressantes	☐	☐	☐	☐	☐	☐

C.2 Dans quelle mesure les affirmations suivantes décrivent-elles vos actions et vos comportements ?

	Pas du tout d'accord	Pas tout à fait d'accord	Plutôt d'accord	Assez d'accord	Tout à fait d'accord	Aucune réponse
Dans des situations difficiles, je peux compter sur mes propres capacités.	☐	☐	☐	☐	☐	☐
Je peux résoudre la plupart des problèmes tout(e) seul(e).	☐	☐	☐	☐	☐	☐
En général, je suis capable de venir à bout des tâches même compliquées et pénibles.	☐	☐	☐	☐	☐	☐

C.3 Au cours des 2 dernières semaines, à quelle fréquence les problèmes suivants vous ont-ils perturbé(e) ?

	Jamais	Durant quelques jours	Durant plus de la moitié des jours	Presque tous les jours	Aucune réponse
Peu d'intérêt ou de plaisir à faire les choses	☐	☐	☐	☐	☐
Être triste, déprimé(e) ou désespéré(e)	☐	☐	☐	☐	☐
Un sentiment de nervosité, d'anxiété ou de tension	☐	☐	☐	☐	☐
Une incapacité à arrêter de s'inquiéter ou à contrôler ses inquiétudes	☐	☐	☐	☐	☐

C.4 De manière générale, dans quelle mesure êtes-vous satisfait(e) de votre vie ? *(0 signifie "pas du tout satisfait" et 10 "tout à fait satisfait")*

pas du tout satisfait … tout à fait satisfait

0	1	2	3	4	5	6	7	8	9	10
☐	☐	☐	☐	☐	☐	☐	☐	☐	☐	☐

C.5 Veuillez indiquer dans quelle mesure vous êtes satisfait(e) des domaines de la vie suivants: *(0 signifie "pas du tout satisfait" et 10 "tout à fait satisfait".)*

pas du tout satisfait … tout à fait satisfait

Êtes-vous satisfait(e) de …	0	1	2	3	4	5	6	7	8	9	10
… votre situation financière?	☐	☐	☐	☐	☐	☐	☐	☐	☐	☐	☐
… vos relations personnelles, familiales et sociales ?	☐	☐	☐	☐	☐	☐	☐	☐	☐	☐	☐
… votre temps libre ?	☐	☐	☐	☐	☐	☐	☐	☐	☐	☐	☐
… votre situation de logement ?	☐	☐	☐	☐	☐	☐	☐	☐	☐	☐	☐
… votre santé en général ?	☐	☐	☐	☐	☐	☐	☐	☐	☐	☐	☐
… votre situation professionnelle ?	☐	☐	☐	☐	☐	☐	☐	☐	☐	☐	☐

C.6 Avez-vous vécu un événement positif au cours des 6 derniers mois?

☐ Oui, à savoir: ____________________ ☐ Non ☐ Aucune réponse

D. Questions sur l’exclusion des jeux

D.1 Faites-vous actuellement l’objet d’une exclusion des jeux de hasard dans un casino suisse ? **Question obligatoire!** Cette question est tres importante. Merci d’y répondre.

☐ Oui ↓ ☐ Non → continuer avec **la question D.4**

D.2 Étiez-vous déjà exclu au moment de la dernière enquête (il y a 6 mois) ? **Question obligatoire!**

☐ Oui → continuer avec **la question D.16**
☐ Non ↓

D.3 Quand l’exclusion a-t-elle été prononcée ? **Question obligatoire!**

______ Date (p. exp. 20.2.2019) → continuer avec **la question D.6**

D.4 Avez-vous été exclu au moment de la dernière enquête (il y a 6 mois) ?

☐ Oui ↓
☐ Non → continuer avec **la question D.17**

D.5 Quand l’exclusion a-t-elle été levée ? **Question obligatoire!**

______ Date (p. exp. 20.2.2019) → continuer avec **la question D.17**

D.6 Quelle offre a été la raison de l’exclusion ? **Question obligatoire!**

☐ Jeu de hasard au casino ☐ Jeu de hazard en ligne ☐ Swisslos, LoRo

D.7 De quel type d’exclusion s’agit-il ? **Question obligatoire!**

☐ Exclusion des jeux imposée ↓
☐ Exclusion des jeux volontaire → continuer avec **la question D.12**

Pour des exclusion des jeux imposées:

D.8 Selon vous, avez-vous été exclu(e) trop tôt, au bon moment ou trop tard ?

☐ Trop tôt ☐ Au bon moment ☐ Trop tard ☐ Aucune réponse

D.9 Pour quelle raison faites-vous l’objet d’une exclusion imposée ?

☐ Je n’ai produit aucune attestation de ma situation financière
☐ Une tierce personne a envoyé une dénonciation
☐ Aucune réponse
☐ Les autorités sociales ont envoyé une notification
☐ Exclusion en raison de tricherie / fraude
☐ Autre raison : ______

D.10 Le fait que votre exclusion du jeu vous ait été imposée vous paraît-il justifié ?

☐ Oui ☐ Non ☐ Aucune réponse

D.11 Pourquoi n’avez-vous pas volontairement demandé à être exclu(e) ? (Veuillez donner uniquement la raison principale)

☐ Aucune raison ne peut justifier une exclusion
☐ Ce n’est pas agréable de demander volontairement à être exclu(e)
☐ Je ne savais pas qu’une telle demande était possible
☐ Je voulais continuer à participer à des jeux de hasard
☐ L’exclusion des jeux est réservée aux personnes dépendantes au jeu
☐ Aucune réponse
☐ Autre raison : ______
☐ Je voulais tout d’abord gagner l’argent que j'avais perdu
☐ Lorsque l’on est exclu(e) des casinos, on peut malgré tout trouver un moyen ou un autre d’accéder aux jeux

→ continuer avec **la question D.16**

Pour des Exclusion des jeux volontaires:

D.12 Vous êtes-vous fait exclure trop tôt, au bon moment ou trop tard ?

- ☐ Trop tôt
- ☐ Au bon moment
- ☐ Trop tard
- ☐ Aucune réponse

D.13 Pour quelle raison avez-vous demandé volontairement à être exclu(e) des casinos ? (Veuillez donner uniquement la raison principale)

- ☐ À titre de préventif
- ☐ Souhait de la famille ou d'ami(e)s
- ☐ Perdu trop d'argent
- ☐ Risqué des mises d'argent trop élevées, sans proportion avec mes revenus ou ma fortune
- ☐ Dettes
- ☐ Problèmes financiers
- ☐ Passé trop de temps au casino
- ☐ Problèmes relationnels ou familiaux
- ☐ Problèmes au travail
- ☐ J'avais perdu le contrôle sur mon activité de jeu
- ☐ Suggestion de la part d'un professionnel
- ☐ Un proche a accepté d'être exclu
- ☐ Je suis dépendant(e) au jeu
- ☐ Aucune réponse
- ☐ Autre raison : ____________

D.14 À quel point votre décision de demander à être exclu(e) a-t-elle été spontanée ? *(1 signifie "absolument pas spontanée" et 10 signifie "complètement spontanée")*

Absolument pas spontanée									Complètement spontanée
1	2	3	4	5	6	7	8	9	10
☐	☐	☐	☐	☐	☐	☐	☐	☐	☐

D.15 Qui a joué le rôle le plus important dans votre décision de demander à être exclu(e) ?

- ☐ Moi-même
- ☐ Mon compagnon / ma compagne
- ☐ Mes proches / mes ami(e)s
- ☐ Le personnel du casino
- ☐ Un(e) conseiller(ère) / thérapeute
- ☐ Autre: ____________
- ☐ Aucune réponse

Pour des des exclusion des jeux volontaires et des exclusion des jeux imposées:

D.16 Imaginons que votre exclusion de jeu est levée : pensez-vous que vous seriez susceptible de rencontrer à nouveau des problèmes liés au jeu ? *(1 signifie "pas du tout susceptible" et 10 signifie "très susceptible")*

Pas du tout susceptible										Très susceptible
0	1	2	3	4	5	6	7	8	9	10
☐	☐	☐	☐	☐	☐	☐	☐	☐	☐	☐

D.17 Le personnel du casino est-il venu discuter avec vous à propos de votre comportement de jeu ?

- ☐ Oui
- ☐ Non
- ☐ Aucune réponse

D.18 Êtes-vous actuellement exclu(e) des jeux de hasard dans un casino à l'étranger ?

- ☐ Oui ↓
- ☐ Non → `continuer avec` **`la question E.1`**
- ☐ Aucune réponse → `continuer avec` **`la question E.1`**

D.19 Dans quel(s) pays faites-vous l'objet d'une exclusion ?

- ☐ Allemagne
- ☐ France
- ☐ Italie
- ☐ Liechtenstein
- ☐ Autriche
- ☐ Autre : ____________

E. Questions sur l’aide et le soutien

Les questions suivantes concernent le recours aux prestations de traitement ou de conseils à cause **des jeux de hasard**.

E.1 Avez-vous bénéficié de prestations d’aide sous forme de conseils ou de traitement en raison de votre pratique des jeux de hasard au course de 6 derniers mois ?

☐ Oui ☐ Non ☐ Aucune réponse

E.2 Avez-vous déjà bénéficié de prestations d’aide sous forme de traitement ou de conseil en raison de votre pratique de hasard ?

☐ Oui ↓

☐ Non, je ne l`ai pas encore → continuer avec **la question E.4**

☐ Non car je n’ai pas de problème de jeu → continuer avec **la question F.1**

☐ Aucune réponse → continuer avec **la question F.1**

E.3 Veuillez indiquer si, au cours des 6 derniers mois, vous avez eu recours à un ou plusieurs des services de conseil ou de traitement suivants à cause des jeux de hasard, et à quelle fréquence approximativement.

Un groupe d’entraide	☐ Pas utilisé	☐ Utilisé :	fois
Un groupe d’entraide sur internet (chat/forum)	☐ Pas utilisé	☐ Utilisé :	fois
Les offres d’information et de conseil sur internet	☐ Pas utilisé	☐ Utilisé :	fois
Un bureau de consultation en matière de dettes	☐ Pas utilisé	☐ Utilisé :	fois
Mon médecin de famille / médecin généraliste	☐ Pas utilisé	☐ Utilisé :	fois
Un(e) psychologue ou un(e) psychiatre	☐ Pas utilisé	☐ Utilisé :	fois
Une hospitalisation à l’hôpital / à la clinique	☐ Pas utilisé	☐ Utilisé :	fois
Consultations ambulatoires spécialisées en addiction	☐ Pas utilisé	☐ Utilisé :	fois
Des parents et/ou amis/amies	☐ Pas utilisé	☐ Utilisé :	fois
Les dignitaires religieux (par exemple : un pasteur, un imam, un rabbin, etc.)	☐ Pas utilisé	☐ Utilisé :	fois
D’autres prestations de soutien	☐ Pas utilisé	☐ Utilisé :	fois

→ **continuer avec la question F.1**

E.4 Pour quelle(s) raison(s) n'êtes-vous pas allé(e) chercher de l'aide ? Veuillez indiquer à quel point les raisons suivantes correspondent à votre situation:

	Pas du tout d'accord	Pas d'accord	Plutôt d'accord	D'accord	Tout à fait d'accord	Aucune réponse
Je ne savais pas où aller trouver de l'aide.	☐	☐	☐	☐	☐	☐
Je m'inquiétais de ce que les autres penseraient de moi.	☐	☐	☐	☐	☐	☐
Je croyais qu'une prise en charge ne m'aiderait pas.	☐	☐	☐	☐	☐	☐
Je pensais qu'une prise en charge me prendrait trop de temps et d'énergie.	☐	☐	☐	☐	☐	☐
Je pensais que je m'en sortirais tout(e) seul(e).	☐	☐	☐	☐	☐	☐
Je ne voulais pas admettre que j'avais besoin d'aide.	☐	☐	☐	☐	☐	☐
J'avais l'impression que le jeu n'était pas une source de problèmes dans ma vie.	☐	☐	☐	☐	☐	☐
J'avais trop de fierté pour aller chercher de l'aide.	☐	☐	☐	☐	☐	☐
Je me sentais incapable de discuter de mes problèmes avec d'autres personnes.	☐	☐	☐	☐	☐	☐
Je ne voulais pas être étiqueté(e) comme une personne dépendante ou malade mentale.	☐	☐	☐	☐	☐	☐
J'ai eu des mauvaises expériences avec les organismes d'aide professionnels.	☐	☐	☐	☐	☐	☐
Ma famille et mes ami(e)s ne m'ont pas suffisamment encouragé(e) à aller chercher de l'aide.	☐	☐	☐	☐	☐	☐
Aucune aide spécialisée en matière de problèmes liés aux jeux de hasard n'était proposée dans mon lieu de domicile.	☐	☐	☐	☐	☐	☐
J'avais peur de me sentir en échec si je ne parvenais pas à décrocher du jeu, malgré l'aide reçue.	☐	☐	☐	☐	☐	☐

F. Questions sur la consommation de substances

Les questions suivantes nous permettent d'avoir un aperçu de votre consommation d'alcool et/ou d'autres substances **au cours des 6 derniers mois**.

F.1 Combien de fois avez-vous consommé des boissons alcoolisées au cours des six derniers mois ?

☐ Jamais → continuer avec **la question F.3**
☐ Moins d'une fois par mois
☐ 1 – 3 fois par mois
☐ 1 – 2 fois par semaine
☐ 3 – 4 fois par semaine
☐ 5 – 6 fois par semaine
☐ Tous les jours

F.2 Lors d'une journée durant laquelle vous consommez de l'alcool, combien buvez-vous en moyenne ?

____ Nombre verre/s de vin (1 verre = 1 dl)
____ Nombre de bière/s (1 bière = 3,3 dl)
____ Nombre de verre/s d'alcool fort (1 verre = 2 cl)

F.3 Combien de fois avez-vous fumé des cigarettes au cours des six derniers mois ?

- ☐ Jamais → `continuer avec` **`la question F.5`**
- ☐ Moins d'une fois par mois
- ☐ 1 – 3 fois par mois
- ☐ 1 – 2 fois par semaine
- ☐ 3 – 4 fois par semaine
- ☐ 5 – 6 fois par semaine
- ☐ Tous les jours

F.4 Au cours d'une journée typique où vous fumez des cigarettes, combien en fumez-vous ?

____ (nombre)

F.5 Combien de fois avez-vous consommé du cannabis au cours des six derniers mois ?

- ☐ Jamais
- ☐ Moins d'une fois par mois
- ☐ 1 – 3 fois par mois
- ☐ 1 – 2 fois par semaine
- ☐ 3 – 4 fois par semaine
- ☐ 5 – 6 fois par semaine
- ☐ Tous les jours

F.6 Combien de fois avez-vous consommé d'autres substances illicites au cours des 6 derniers mois ?

- ☐ Jamais
- ☐ Moins d'une fois par mois
- ☐ 1 – 3 fois par mois
- ☐ 1 – 2 fois par semaine
- ☐ 3 – 4 fois par semaine
- ☐ 5 – 6 fois par semaine
- ☐ Tous les jours

→ Quelles substances? ____

Vos commentaires, remarques ou questions concernant l'étude, les jeux de hasard en général ou autres sont les bienvenus dans le champ ce saisie.

____

Votre code personnel

Afin de pouvoir attribuer les données des trois questionnaires à une personne et de préserver leur anonymat, nous utilisons un code anonyme au lieu de votre nom. Personne ne connaît ce code, sauf vous. Vous n'avez pas besoin de vous souvenir du code. Nous vous demanderons de créer le même code pour la 3ème enquête.

Ce code personnel est constitué d'une combinaison de lettres et de chiffres.
Veuillez entrer …

- … la dernière lettre du mois de votre naissance (p. ex. : janvie**r**).
- … les deux premières lettres du prénom de votre mère (p. ex. : **An**na).
- … les deux premières lettres du prénom de votre père (p. ex. : **Ma**rk).
- … les troisième et quatrième chiffres de votre année de naissance (p. ex. : 19**79**).

→ De l'exemple résulte le code « ranma79 »

Veuillez saisir votre code ici: ____

Merci pour votre participation! – L'équipe de l'étude sur le jeu
